# Physical fitness characteristics of elite freestyle skiing aerials athletes

**DOI:** 10.1371/journal.pone.0304912

**Published:** 2024-06-06

**Authors:** Youwei Yao, Xuesong Niu

**Affiliations:** 1 School of Sports Training, Shenyang Sport University, Shenyang, China; 2 School of Social Sports, Shenyang Sport University, Shenyang, China; University of Montenegro, MONTENEGRO

## Abstract

**Objective:**

To analyze the physical fitness characteristics of elite freestyle skiing aerials athletes, thereby enhancing the understanding of exercise physiologists, sports scientists, and coaches regarding the demands in this discipline.

**Methods:**

After health screenings, 29 athletes from the Chinese National Freestyle Skiing Aerials Team were divided into elite and general groups, including males and females. Physical fitness indexes were determined through literature reviews, expert interviews, and the Delphi method, followed by physical fitness tests assessing body morphology, physiological function, and physical quality. Data normality was verified using the Shapiro-Wilk test. Differences between the two groups were then evaluated using independent sample t-tests or Mann-Whitney U tests, after which effect sizes were calculated to assess the magnitude of the differences.

**Results:**

Significant body morphology differences were noted between elite and general groups in fat-free body weight, leg, and waist circumferences (P < 0.05). Male athletes in the elite group exhibited a significantly lower percentage of body fat (P < 0.05), whereas the reduction in body fat percentage among female elite athletes was not statistically significant. In terms of physiological function assessment, elite athletes demonstrated superior performance in both maximum anaerobic capacity and relative maximum anaerobic capacity compared to their counterparts in the general group (P < 0.05). Notably, the difference in maximum anaerobic capacity was highly significant among male athletes (P < 0.01), and the relative maximum anaerobic capacity among female athletes was also markedly significant (P < 0.01). Regarding physical quality indexes, elite athletes outperformed those in the general group in all aspects except for the quick v-up and 12-minute run tests (P < 0.05 or P<0.01).

**Conclusion:**

Elite athletes exhibit superior physical fitness characteristics compared to general athletes, attributable to differences in age, years of training, and their participation in ongoing specialized physical training within structured, cyclical programs. Specifically, elite athletes demonstrated higher fat-free body weight, larger waist and leg circumferences in terms of body morphology. Particularly, male athletes showed a trend towards lower body fat percentage. Physiologically, they exhibited stronger anaerobic metabolism capabilities. In terms of physical quality, elite athletes displayed superior limb strength, lower limb explosive power, and specialized core strength, along with better speed, agility, and overall coordination.

## Introduction

Freestyle skiing aerials, a highly challenging winter sport, originated in the United States in the 1960s. It was officially recognized by the International Ski Federation (FIS) in 1979 and included in the Winter Olympics in 1994. China began developing this sport in the late 1980s, and it has since become a key strength in the country’s Winter Olympics lineup. At the 24th Beijing Winter Olympics in 2022, the Chinese freestyle skiing aerials team achieved their best historical result with 2 golds and 1 silver, demonstrating their strong competitive prowess.

Stability, difficulty, accuracy, and aesthetics are the main competitive factors and distinctive characteristics of freestyle skiing aerials [1]. The sport encompasses a range of technical maneuvers, spanning from single movements to more complex double and triple movements. As the global competitiveness of freestyle skiing aerials advances, high-difficulty triple movements have become crucial for medal contention (current mainstream triple movements for females include bLFF, bLTF, bFFF, bLdFF, and for males, bdFFdF and bFdFdF). The sport consists of four phases [2, 3]: inrun, takeoff, aerial maneuver and landing. Judges score the athletes’ technical maneuvers. The score for each maneuver is then multiplied by the corresponding difficulty coefficient, determining the final score for each maneuver. Scoring is divided with 20% for air, 50% for form, and 30% for landing [4]. Typically, the greater the difficulty of the maneuvers executed by athletes, and the better the quality of the execution, the higher the probability of winning [5].

Aerials athletes are required to perform complex techniques involving flips and rotations during competitions or training, which place high demands on their execution control, and also tend to induce fatigue [6–8]. The takeoff and aerials demand acute spatiotemporal perception, core stability and power output, and precise neuromuscular control [9]. Landing requires strong eccentric lower limb strength and control of balance stability [10, 11], with failure not only affecting scores but also increasing injury risks [12, 13]. Among Winter Olympic sports, the freestyle skiing aerials event has a notably high injury incidence rate of 28.6%, with non-contact knee injuries being common [14]. The high rate of injuries is primarily due to the sport involving extreme performances and challenging terrain structures, such as the unstable landing slope, which disrupts athletes’ body perception and increases risks during landing. After takeoff, the vertical drop to the landing spot often exceeds 10 meters and can potentially reach or surpass 18 meters in height difference [9, 15]. During the landing phase of triple movements, the impact force on the soles of the feet averages 7.06 to 8.13 times the body weight [16]. In addition to the stress transmitted along the long axis of the lower limbs, different loads on the skis during landing exert anterior-posterior and rotational stresses on the knee joint [15]. This increases the risk of injury to the joint cartilage, menisci, subchondral bone, and ligaments. Locking athletes’ ankles in ski boots diminishes cushioning and amplifies impact forces on knee joints during landing [17]. The cold and variable outdoor environment further increases the risk of injury to athletes.

Strength training is an essential component of physical fitness training for freestyle skiing aerials athletes, crucial for enhancing athletic performance and reducing both acute and chronic injuries [9, 18]. The enhancement of specific strength, particularly in the hip, knee, ankle joints, and trunk muscles, is key for performing high-quality technical maneuvers and ensuring safe landings [19]. Improving core endurance and the rapid concentric contraction ability of the hip flexors significantly reduces spinal and knee injuries, while also optimizing landing techniques [10]. Enhancing core strength and balance helps reduce the bending angles of the hips and knees upon landing, diminishes fluctuations along the horizontal axis, and effectively counters post-landing inertial impacts [10, 20]. Targeted strength training also accelerates the rehabilitation process for injured athletes, facilitating a quicker return to competition [21]. Additionally, the extended duration of training and competitions, often lasting 2–4 hours with intervals of over 5 minutes between each technical move, places significant demands on athletes’ recovery capabilities, highlighting the need for robust physical conditioning [18, 22].

Athletic physical fitness increasingly determines competition outcomes [23]. Globally, especially in winter sports powerhouses, research on physical fitness, including anthropometry [24–27], metabolic characteristics [28–31], and athletic abilities [32–35], is extensive. However, there remains a lack of comprehensive and quantitative research on the physical characteristics of freestyle skiing aerials athletes, and no study has compared the physical differences between aerials athletes at different ability levels. In freestyle skiing aerials, athletes are challenged to excel in both physical fitness and technical skills. Understanding the specific physical requirements for top-level aerials competitors allows for more effective training strategies to develop and enhance these attributes in athletes. This approach is crucial for advancing performance standards in this demanding sport. Therefore, this study collected data on the three aspects of body morphology, physiological function, physical quality and systematically and comprehensively analyzed the physical characteristics of China’s elite aerials athletes. Our aim is to motivate a deeper comprehension among sports physiologists, sports scientists, and especially coaches, of the requirements needed for proficiency in the aerials event of freestyle skiing. This understanding is crucial for the development of comprehensive protocols and strategies that effectively assess the physical attributes of aerials athletes. Furthermore, it aids in monitoring their performance, offering pragmatic training guidance, and devising precise talent identification program.

## Methods

### Participants

This study involved 29 athletes from the national freestyle skiing aerials team, who were actively preparing to compete in the 2022 Beijing Winter Olympics. The athletes were systematically classified into two distinct groups based on their performance levels in national and international competitions. The Elite Group, comprising 6 men and 5 women, consists of international master-level athletes who have achieved top-three finishes in major events such as the Winter Olympics, World Championships, or World Cups. The General Group includes 9 men and 9 women from various provincial teams across China, with achievements up to top-three finishes in national competitions. Further details about these groups are provided in Table 1.

**Table 1:** Basic information of participants.

			Calendar Age (yrs)		Training age (yrs)		Height (cm)		Weight (kg)	
Group	Sample (n)		Male (M± SD)	Female (M± SD)	Male (M± SD)	Female (M± SD)	Male (M± SD)	Female (M± SD)	Male (M± SD)	Female (M± SD)
Elite Group	5	6	23.48±4.40	24.61±4.20	10.56±4.32	10.20±5.61	174.15±2.14	161.90±3.94	68.57±3.51	56.22±3.52
Excellent Group	9	9	19.56±2.29	20.22±3.67	7.85±3.61	8.34±4.59	175.53±4.99	158.78±3.46	69.21±4.82	55.37±5.92

### Study design

To prepare for this study, we reviewed and organized literature related to freestyle skiing aerials and similar technical sports, encompassing both print and online materials. This helped us gain a comprehensive understanding of the current research landscape and recent advancements in this field. On this basis, this paper determined the research framework.

To delve into the specific traits of aerials event along with the athletes’ physical fitness, and to ensure the scientific rigor, practical application, and feasibility of our study, we consulted experts in this discipline from universities such as Beijing Sport University and Shenyang Sport University (a total of 6 experts), as well as coaches from national and provincial aerials teams (a total of 10 coaches). Through face-to-face interviews and telephone conversations, we sought expert advice on aspects including physical fitness test index selection, testing specifics, and evaluation methodologies (S1 Appendix).

The first two steps in this study were determining the primary selection indexes for the physical fitness test and designing the questionnaire (S2 Appendix). This survey was carried out from April to May 2020 with a group of 12 experts, including aerials coaches, referees, and researchers, who conducted a two-stage survey to assess the importance of these indexes. A 5-point Likert scale was utilized for the questionnaire, ranging from “very inappropriate” to “very appropriate” for rating each index. This methodological approach aimed to identify the crucial physical fitness parameters for elite athletes in this discipline. According to the scores and comments of the first round of expert questionnaires, some indexes were deleted or modified, and then the second round of questionnaires was developed. The coefficient of variation (CV) indicated the degree of difference between experts in understanding of the relative importance of a particular index. Under normal circumstances, CV<0.25 is considered to be an acceptable range. The smaller the CV was, the higher the degree of coordination between experts on that index [36–38]. After the second round of questionnaires, we chose the indexes with CV<0.25 as the final test indexes. Each of the 12 questionnaires issued in the survey rounds was returned.

The questionnaire’s effectiveness was affirmed, with over 90% of experts expressing satisfaction with its structure and content (refer to S3 Appendix). To assess the questionnaire’s reliability, a retest method was employed. Six participants from the initial survey were randomly chosen for a follow-up survey two weeks later. Analysis of both survey results using statistical software yielded a retest reliability coefficient of 0.87, indicating the questionnaire’s high reliability in measuring physical fitness evaluation indexes.

### Procedures and protocols

In June 2020, we recruited athletes as participants for this study and conducted a week-long series of tests within the same month. Before the commencement of the physical fitness tests, medical professionals screened the health conditions of the participants to ensure all involved athletes were in excellent physical health and free from any contraindications that could affect competitive training, such as cardiovascular diseases or musculoskeletal injuries. Out of 30 national team aerials athletes, 29 met our inclusion criteria. All participants voluntarily signed a written informed consent, confirming their willingness to participate in this study. This study was approved by the Ethics Committee of Shenyang Sport University (approval number: 10096) and strictly adhered to the ethical standards of the Declaration of Helsinki. Prior to the tests, athletes engaged in standardized warm-up activities. Throughout the testing phase, none of the athletes suffered any injuries. Under the supervision of coaches and scientific researchers, they successfully completed all the testing components required by the study. The athletes underwent body morphology and physical quality tests in the physical fitness training hall and on the track field, while physiological function tests were conducted in the Functional Testing Center by scientific personnel.

For assessing body morphology of freestyle skiing aerials athletes, our approach adhered to the guidelines specified in “Sports Measurement and Evaluation” [39]. The assessments were conducted using calibrated equipment by trained professionals. We employed the InBody270 (Yinbadi Co., Ltd., CN), a multifrequency bioelectrical impedance analyzer, for detailed body composition analysis.

Physiological functions of the athletes were measured using a suite of specialized equipment. This included a MONARK837 anaerobic power bike (Switzerland) for assessing anaerobic capabilities, a MAXII exercise cardiopulmonary function test system (USA) for cardiopulmonary evaluation, and a MONARK839E bicycle ergometer (Switzerland) for endurance testing. Additional physiological and biochemical measurements were undertaken using a range of diagnostic tools, such as the XF-IB hemoglobin analyzer, BT-1904C blood urea biochemical analyzer, BECKMAN STKS red blood cell analyzer, DSL-10-67100 cortisol analyzer, and DSL-10-4000 serum testosterone analyzer, ensuring comprehensive assessment.

To evaluate physical quality of the athletes in aerials event of freestyle skiing, we utilized various equipment like Zhang Kong barbell plates and bars, Jia You sports and leisure products like medicine balls, logo discs, balance pads, yoga mats, and Great Wall Seiko Industrial tapes. The WaterRower ribbed wooden frames were instrumental in assessing strength endurance. Precise timing of the athletes’ performance was recorded using SEIKO electronic stopwatches and the SmartSpeed optical door timing system. Detailed methodologies of these tests are elaborated in the S4 Appendix.

### Data analyses

In this study, physical fitness test data were organized using Microsoft Excel. Descriptive statistical analysis was performed with SPSS 22.0. The Shapiro-Wilk test was used for assessing data normality. The independent sample t-test was applied to normally distributed indexes, and the Mann-Whitney U test was used for those not adhering to a normal distribution, in order to compare differences between two groups of athletes.

To quantify the practical significance of differences between groups, this study calculated the effect size of relevant variables. For variables confirmed to follow a normal distribution by the Shapiro-Wilk test, we calculated the Cohen’s d coefficient to assess the effect size between two independent samples. The effect size for the d coefficient was defined as: small (0.20≤d<0.50), medium (0.50≤d<0.80), and large (d≥0.80) [40, 41]. For variables not conforming to normal distribution, we used the r value to indicate effect size. The general standards for the effect size of the r value are: small (0.10≤r<0.30), medium (0.30≤r<0.50), and large (r≥0.50) [41].

In all statistical analyses, two-tailed tests were used, and the significance level was set at 0.05. In the statistical results, a single asterisk (*) indicates P<0.05, and a double asterisk (**) indicates P<0.01. Moreover, “d” represents the Cohen’s d coefficient value, and “r” represents the r value. For variables adhering to a normal distribution, the presentation of data utilizes means and standard deviations, denoted as M±SD. Conversely, for those variables not following a normal distribution, the data is represented using medians and interquartile ranges, indicated as Median [Q1-Q3] (for detailed information, refer to S5 Appendix).

## Results

### Body morphology test results

**Fig 1:**
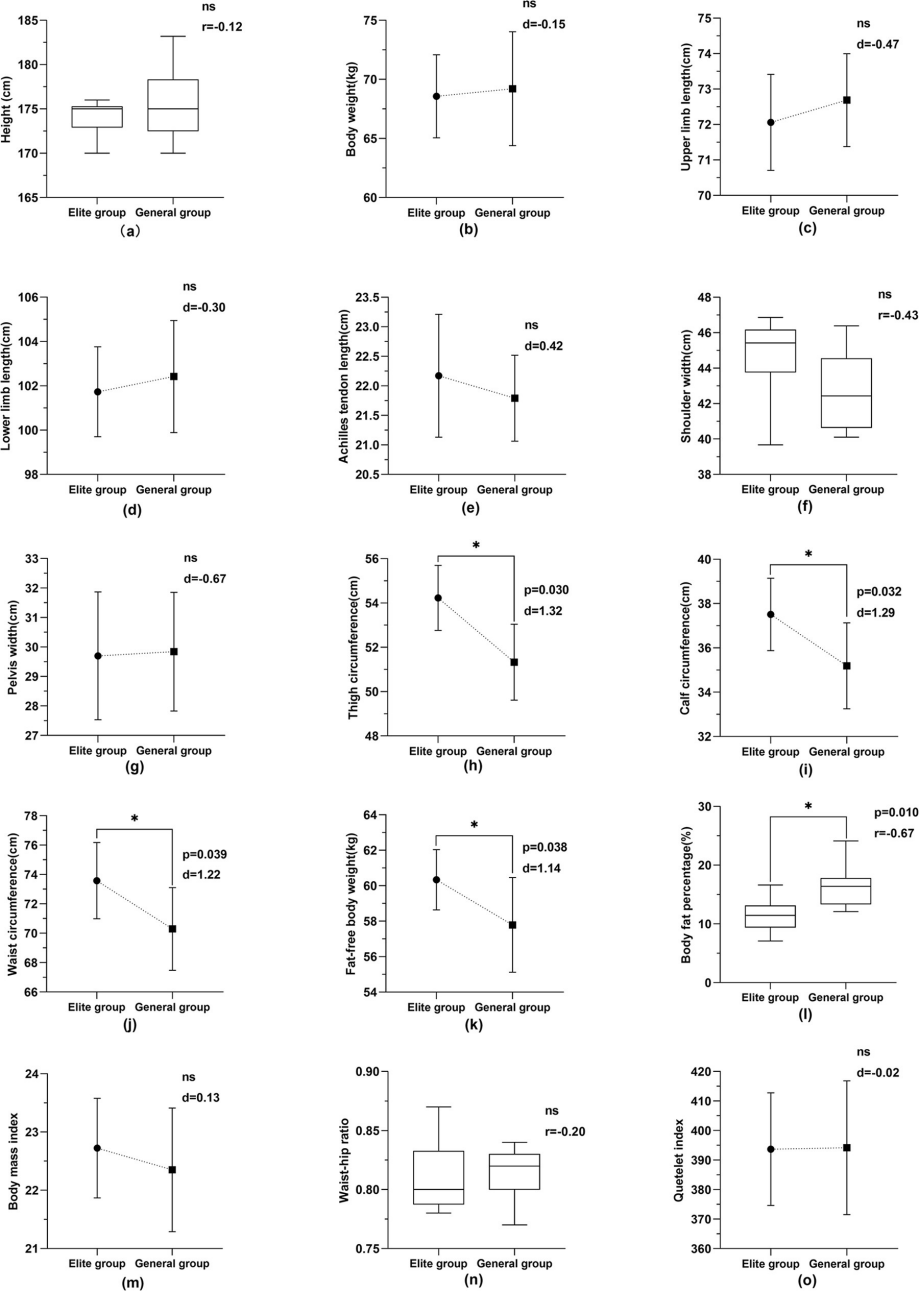
Comparison of male athletes’ body morphology indexes between elite and general groups.

**Fig 2:**
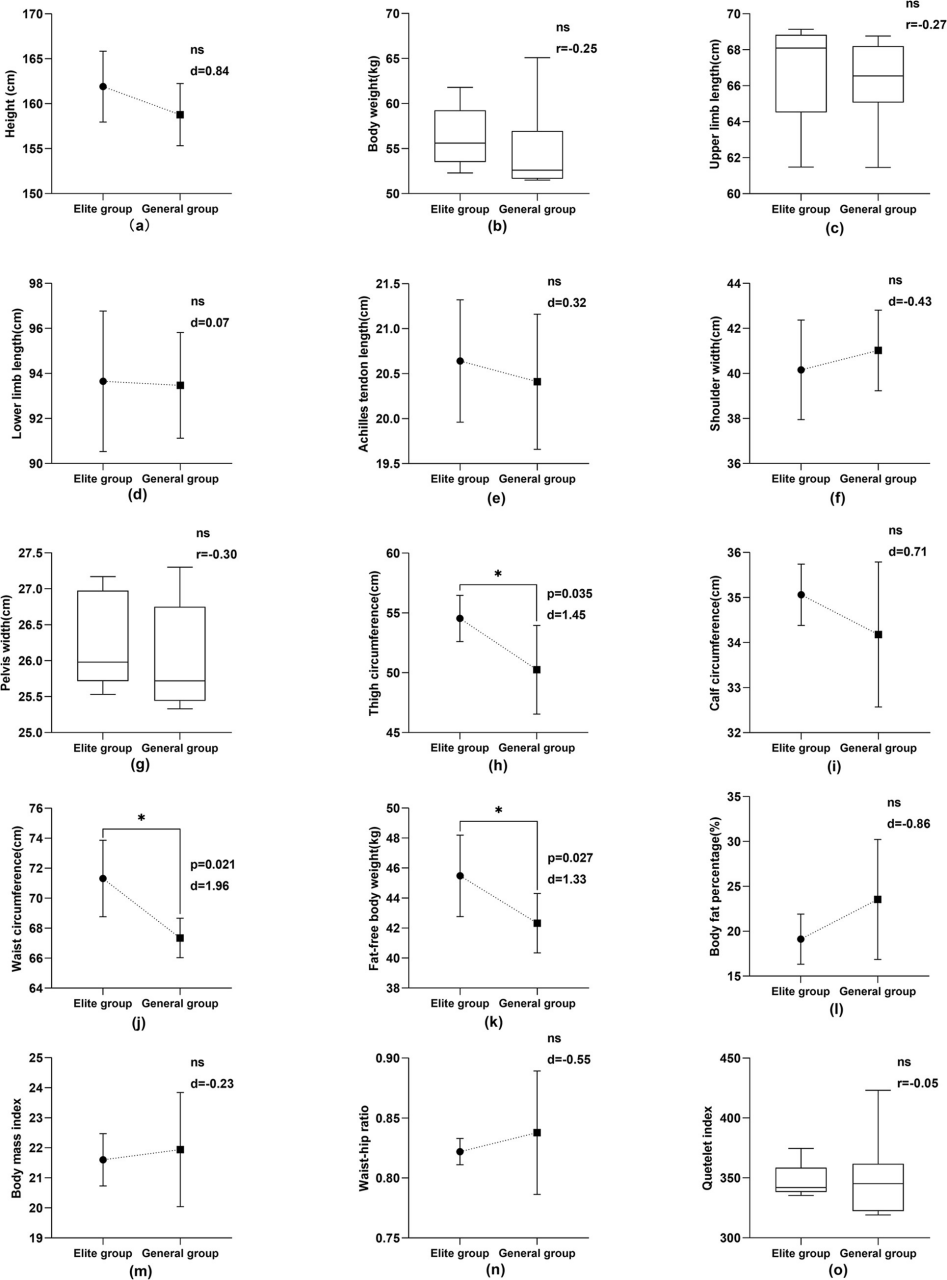
Comparison of female athletes’ body morphology indexes between elite and general groups.

As seen from Figs 1 and 2, among male athletes, significant statistical differences were observed between elite and general athletes in terms of fat-free body weight (p = 0.038, d = 1.14), waist circumference (p = 0.039, d = 1.22), thigh circumference (P = 0.030, d = 1.32), body fat percentage (p = 0.010, r = -0.67), and calf circumference (p = 0.032, d = 1.29). Among female athletes, elite athletes differed significantly from general athletes in fat-free body weight (p = 0.027, d = 1.33), waist circumference (p = 0.021, d = 1.96), and thigh circumference (p = 0.035, d = 1.45). No significant differences were observed in other body morphology indexes between the two groups (P>0.05).

### Physiological function test results

**Fig 3:**
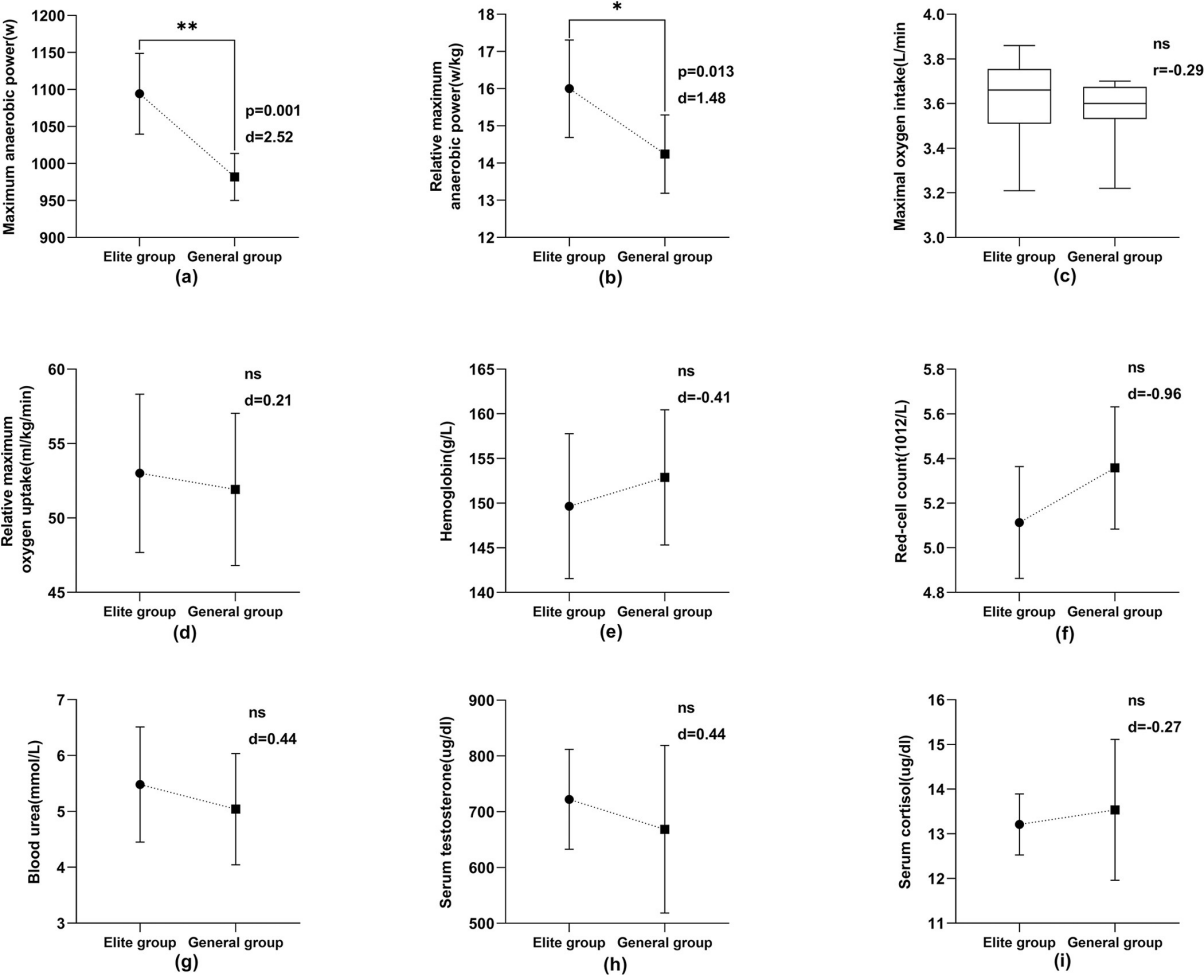
Comparison of physiological function indexes of male athletes in elite and general groups.

**Fig 4:**
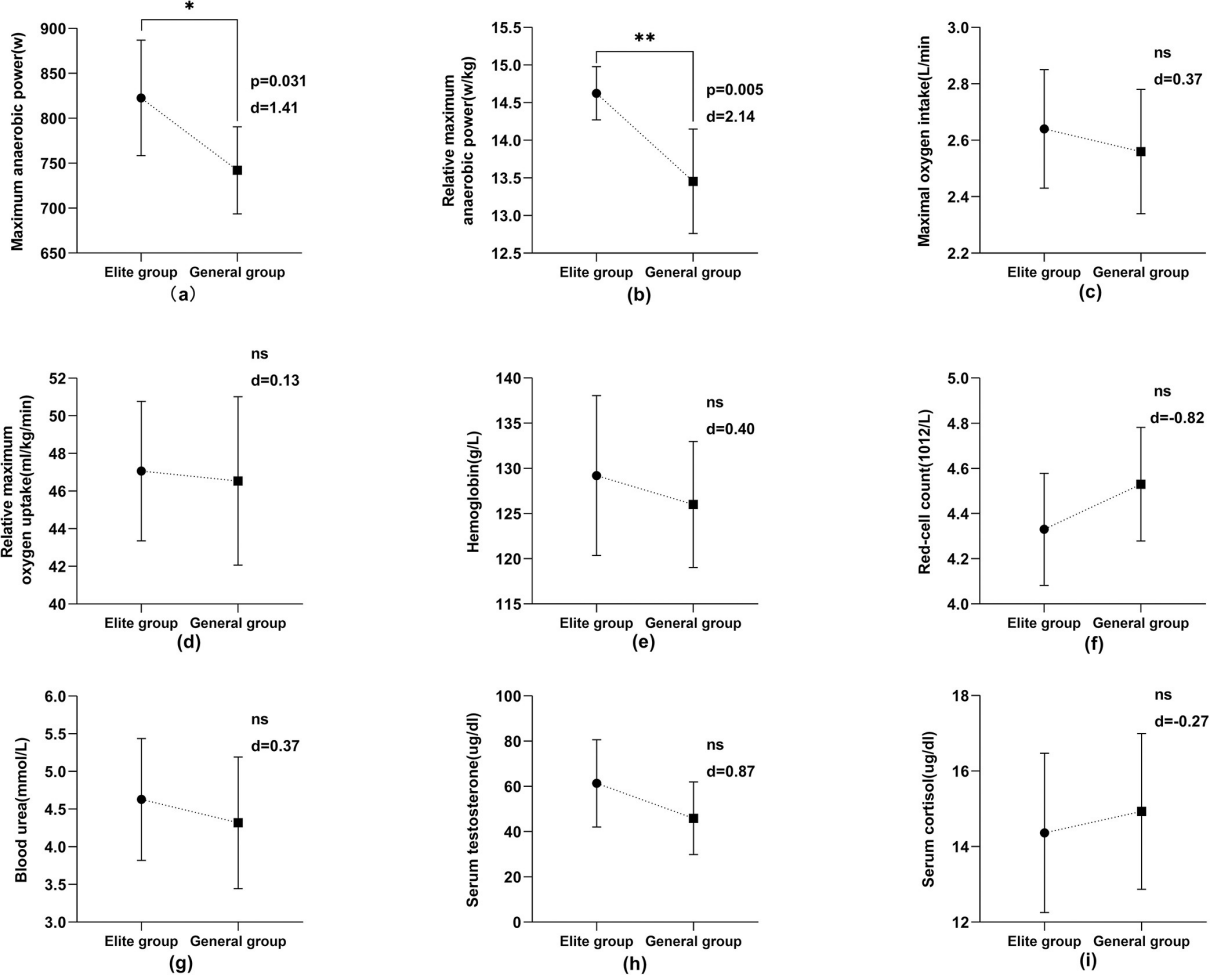
Comparison of physiological function indexes of female athletes in elite and general groups.

According to the statistics of the test data (Figs 3 and 4), the maximum anaerobic power and relative maximum anaerobic power of the elite athletes were significantly higher than those of the general athletes (P<0.05). The maximum anaerobic power (p = 0.001, d = 2.52) of the male athletes was very significantly different (P<0.01), and the relative maximum anaerobic power (p = 0.005, d = 2.14) of the female athletes was very significantly different (P<0.01). The statistical results of other indexes were not statistically significant (P>0.05).

### Physical quality test results

**Fig 5:**
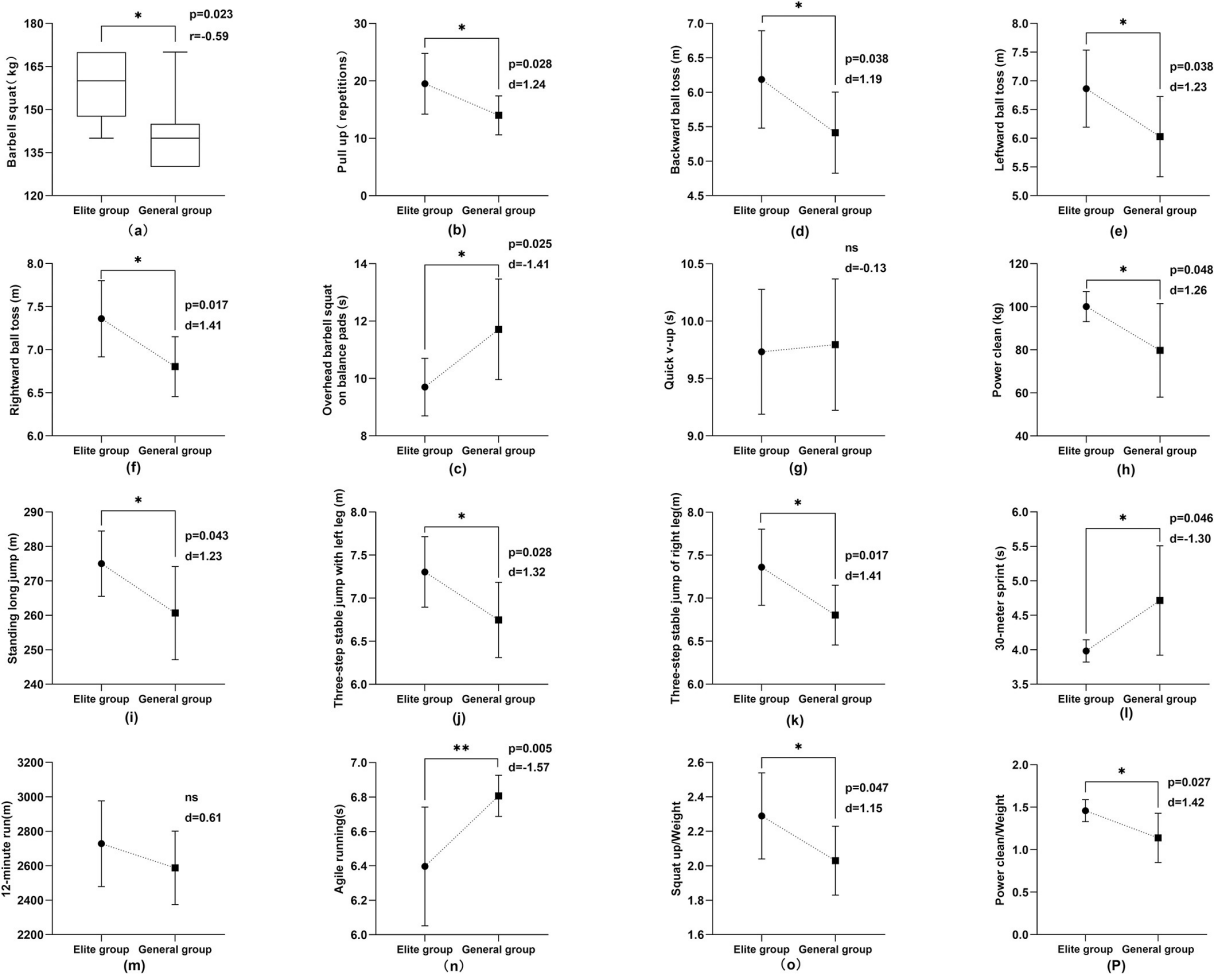
Comparison of physical quality indexes of male athletes in elite and general groups.

**Fig 6:**
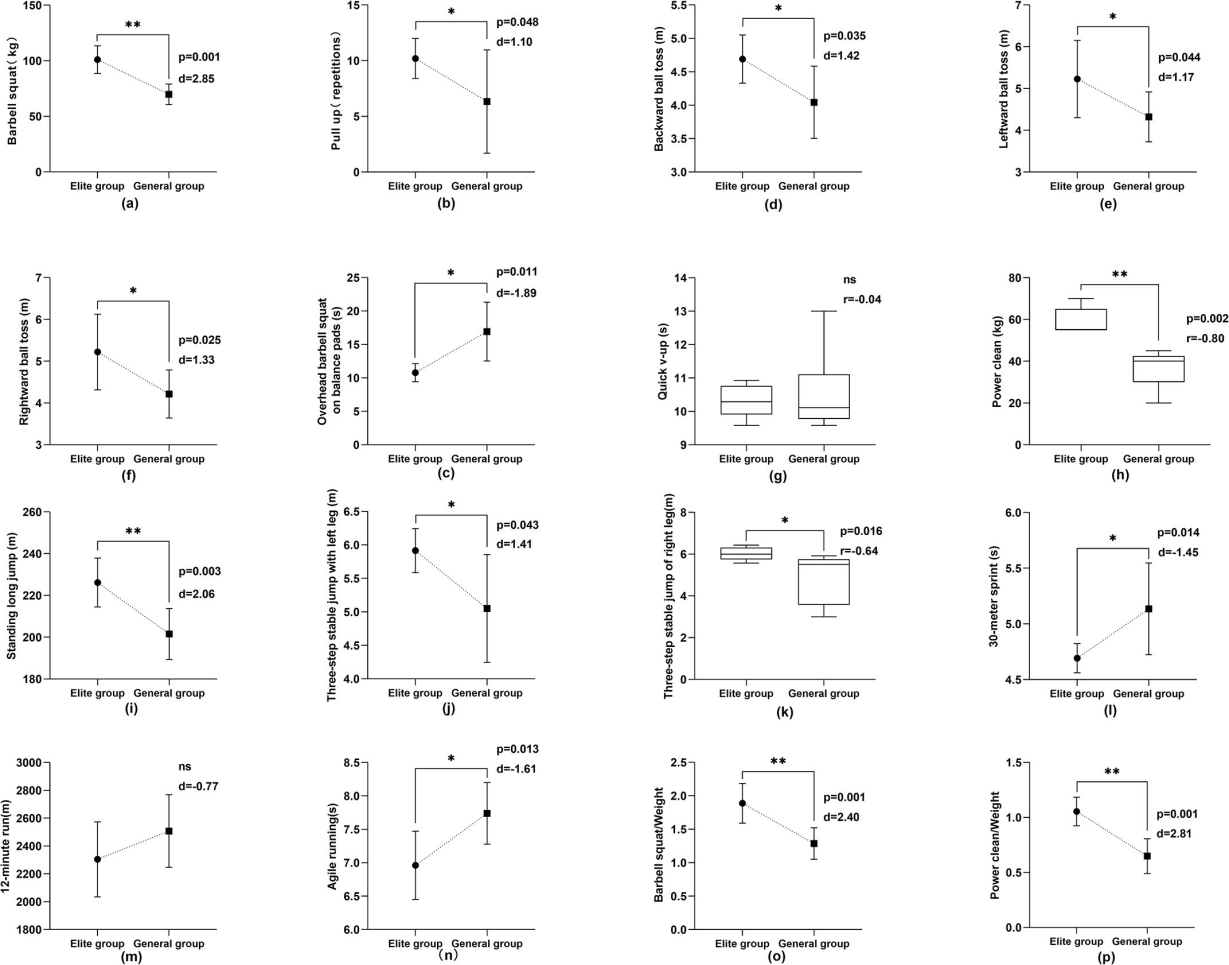
Comparison of physical quality indexes of female athletes in elite and general groups.

As shown in Figs 5 and 6, the other indexes of physical quality in the elite group were better than those in the general group (P<0.05 or P<0.01), excluding the indexes of quick v-up and aerobic endurance.

## Discussion

### Body morphology characteristics

Body composition proportions influence athletic techniques and strategies in competition, as evidenced by studies [42, 43]. The body composition of winter athletes is related to their competition results [26]. Fat-free body weight is made up of all other body components excluding fat tissue, with muscle being the main component. Fat-free body weight affects the athletic ability of athletes [44, 45]. The proportion of fat weight to body weight is called body fat percentage. The fat-free body weight is inversely proportional to the percentage of body fat. The higher the fat-free body weight of an athlete is, the lower the body fat percentage, and vice versa. Figs 1 and 2 show a significant difference between the fat-free weight of male and female athletes in the elite group and that of male and female athletes in the general group (P<0.05). In the comparison of body fat percentage among male athletes, the elite group demonstrated a significantly lower body fat percentage compared to the general group (P < 0.05). In contrast, while the female elite athletes did not differ significantly from the general group in terms of body fat percentage statistically, the mean value for the elite group was still lower. These findings suggest that elite athletes tend to have a physique characterized by higher fat-free body weight and lower body fat content, a trend that is particularly evident in male athletes. According to the characteristics of aerials, strength is the core physical quality of the event [23]. The body’s exercise ability and power level are closely related to fat-free body weight [19, 45]. The higher the proportion of fat-free body weight, the stronger the muscle strength and explosive power are. Therefore, athletes with a high proportion of fat-free weight and low body fat content are better able to complete complex technical movements and meet the needs of the competition.

The study unveiled significantly larger thigh and waist circumferences in elite athletes compared to their general counterparts (P<0.05), with elite male athletes showing notable differences in calf circumference (P<0.05). These elite athletes, having low body fat content and larger waists and legs, display higher strength levels. The large waist circumference of elite athletes results from long-term specialized training, reflecting the high degree of development of their waist and abdominal core muscles. The unique techniques of aerials have very high requirements for the stability and power output of athletes’ core muscles. On one hand, the solid lumbar pelvic belt forms an overall regional muscle group that ensures that the athlete’s somersault with twist remains stable and prevents deformation while stabilizing the body’s center of gravity. On the other hand, the technical movements of the landing phase are overloaded on the human waist. If there is no strength in the waist and abdominal core muscles to fix the spine, stabilize the pelvis, reduce the impact force, and improve the stability of the landing posture, the probability of injury in athletes will be increased. Therefore, the strength of the waist and abdominal muscles is indispensable for aerials athletes [10]. Furthermore, the significant thigh circumference in elite athletes signifies their prominent lower limb strength. Given that many movements in the aerials event involve lower limbs, strong lower limbs facilitate takeoff and landing [11, 46]. Additionally, developed leg muscles help alleviate knee joint pressure during high-altitude landings, reducing injury risks [47, 48].

### Physiological function characteristics

#### Anaerobic capacity characteristics

Anaerobic metabolic capacity is defined as the physiological capacity for energy production in the absence of oxygen, primarily during high-intensity exercises. It represents the ability of the body to work continuously using the energy supply system of adenosine triphosphate-creatine phosphate (ATP-CP) and glycolysis. Compared with aerobic metabolism, anaerobic metabolism is characterized by a higher intensity but shorter duration. Generally, to master the ability of the body to produce high power output in the shortest time under anaerobic conditions, that is, the ability to exert maximum strength and speed, scientific researchers use an anaerobic power bicycle and the Wingate anaerobic power test method to obtain data [49, 50]. According to previous research results and the characteristics of **freestyle skiing aerials**, athletes need to have good anaerobic metabolism and aerobic metabolism to adapt the energy system of the body to the requirements of this sport, have a good rhythm of energy output and recovery and make adequate preparations for adapting to high-intensity training and competition. The development of anaerobic metabolism is significant for **aerials athletes**.

This study measured maximum anaerobic power and relative maximum anaerobic power in athletes. These two indexes mainly reflect the ability of athletes to do work quickly under the ATP-CP system. These results (Figs 3 and 4) indicated that the two test indexes reflecting anaerobic metabolic ability were significantly higher than those of general athletes (P<0.05). The capability of the ATP-CP energy supply system of elite athletes was better than that of general athletes, and the anaerobic metabolism of elite athletes was more prominent. At the same time, these results also indicate that maximum power and relative maximum power are the typical indexes to evaluate the physiological function level and anaerobic metabolic ability of athletes in this event. After a comparative analysis of the data above, it is evident that the exceptional Chinese aerials athletes possess a strong power supply capacity in the ATP-CP system, a characteristic feature of their bodily function. This capacity enables them to exhibit remarkable movement speed and instant explosive ability.

#### Aerobic capacity characteristics

Maximum oxygen uptake refers to the maximum amount of oxygen taken in and utilized by the body in a unit of time when the cardiovascular system is fully mobilized. Cardiopulmonary function reaches the body’s limit. Maximum oxygen uptake is an important index for evaluating aerobic endurance, manifested as the absolute and relative values. For competitive sports with weight control requirements, the research significance of relative maximum oxygen uptake is greater than that of maximum oxygen uptake [51–53]. As demonstrated in Figs 3 and 4, elite Chinese athletes exhibited higher overall mean values for maximum and relative maximum oxygen uptake compared to the general athlete group, but these differences were not statistically significant (P>0.05).

According to relevant research and analysis, freestyle skiing aerials is a skill-dominated sport with difficult and beautiful performances. The time of muscle movement is short, and the energy consumption from a single movement is small. The impact on vegetative function is poor compared with other sports, so the level of cardiopulmonary function is not prominent [54]. Aerobic metabolism is not the most critical part of the competitive ability of aerials athletes. From the overall point of view of energy metabolism of the body, although the technical movements displayed by the athletes during the competition are fast-paced and short in time, athletes need a good anaerobic metabolic system to provide the energy supply. This does not mean that the coaches overemphasize the training of anaerobic metabolic ability while ignoring aerobic metabolism training, because anaerobic ability needs to be based on aerobic ability to highlight the level of sports technique [18].

#### Physiological and biochemical characteristics

Figs 3 and 4 show that the levels of specific blood biochemical indexes in athletes of different levels are all within the normal ranges [55]. Hemoglobin levels are 120–160 g/L for males and 110–150 g/L for females. Serum testosterone ranges from 270–1070 ng/dl in males and 10–100 ng/dl in females. Blood urea is typically between 1.7–8.3 mmol/L for both genders. The red-cell count is 3.5–5.6 × 10^12/L, and cortisol levels are 5–25 μg/dl for both males and females. Statistical analysis revealed no significant differences across these indexes at different levels of athletes (P>0.05). This indicates that the physiological functions of freestyle skiing aerials athletes at all levels are better, and differences in their biochemical characteristics are minor.

### Physical quality characteristics

#### Strength characteristics

Strength is the foundation for developing other physical qualities and the essential quality that allows athletes to master skills and tactics and improve performance [56]. In aerials techniques, takeoff, aerial maneuvers, and landing techniques are inseparable from the support of strength. Barbell squat reflects the athlete’s lower limb muscle strength, especially the comprehensive strength of the gluteal leg muscle group. It also tests the athlete’s rigid stability in the waist and abdomen region, that is, its static contraction ability. Pull-ups mainly measure the strength and endurance level of the back muscle group and upper limb muscle group of aerials athletes. To achieve elite performance in this index test, athletes need to have a strong relative strength level and speed endurance ability of continuous contraction. As shown in Figs 5 and 6, at present, China’s elite aerials athletes have a stronger level of limb strength than general athletes (P<0.05).

Standing long jumps mainly test the explosive force of the lower limbs. The power clean movement mainly reflects the explosive force of the athlete’s lower limb three-joint extension and overall coordination ability [57]. The three-step stable jump index test evaluates the lower limbs’ ability to initiate and buffer movements quickly, and react to changes, while also assessing the symmetry of lower limb strength to ensure balanced muscle development. Figs 5 and 6 show that compared with general athletes, the athletes in the elite group had significant differences in the above indexes (P<0.05). This indicates that the overall lower limb explosive power level of the athletes in this event plays a vital role in improving their competitive ability.

The definition of *relative strength* is the maximum strength of an athlete per kilogram of body weight, which reflects the relationship between absolute strength and body weight [58]. The relative strength level is essential for freestyle skiing aerials, gymnastics, diving, and other technical sports. This kind of Olympic event requires athletes to have greater maximum strength, and it also requires that athletes not be too heavy. Therefore, athletes need to have good relative strength; that is, under the condition of small weight change, athletes must have a higher maximum strength ability, which is more conducive to the effective completion of their technical actions.

Freestyle skiing aerials is a competitive event with relatively strict weight control [53]. Athletes are affected by wind resistance and other environmental factors when performing maneuvers such as somersaults. Excess weight may lead to reduced agility and smoothness in their movements. In addition, the impact on the knee joint is greater when landing, and the risk of injury will increase [17]. In summary, the weight of aerials athletes must be controlled within the ideal range. Therefore, testing and analysis of the relative strength ability of aerials athletes are more reasonable and targeted. Figs 5 and 6 show that the relative strength indexes of male elite athletes significantly differ from those of the male general athletes (P<0.05) and that the relative strength indexes of female elite athletes exhibit even more significant differences from those of the female general athletes (P<0.01). These results show that a better relative strength level is a typical physical feature of freestyle skiing aerials athletes in China.

Freestyle skiing aerials require athletes to have robust core muscles, evident in testing measures such as overhead barbell squat on balance pads, quick v-up, and different directions of ball tosses. These tests reflect core muscle stability and explosive strength levels, fitting into the category of specialized strength [21]. The overhead barbell squat on balance pads test evaluates core stability under weight-bearing imbalance, and flexibility of hips, knees, ankles, shoulder joints, and spine extension. Quick v-up tests fast coordinated contraction ability of athletes’ core muscles. Backward ball toss measures the instantaneous explosive force of the trunk in backward extension. The leftward and rightward ball tosses evaluate the explosive rotational force of the side core muscles, indicating an athlete’s rotational capacity. Additionally, these tests assess the symmetry of trunk rotational strength. As shown in Figs 5 and 6, in addition to the quick v-up, the core strength test results of the elite group were significantly different from those of the general group (P<0.05). In the aerial maneuvers phase of the technical movement of aerials, the athletes rely on the controlled force of the trunk to generate rotation of the body, transmit the rotation force, promote the coordination of the four limbs to cooperate with the somersault technique, and finally achieve the goal of accurately completing the technical action [2, 59]. A strong core muscle group stabilizes and supports the body posture and unique sports skills of aerials athletes. Maintaining a beautiful upright posture, precise body control, and balance in the air is beneficial [60]. In the landing phase of the technical action, after the athletes finish multiple types of difficult aerial maneuvers, they land on the soft and slippery sloping snow. Even a slight deviation in their body’s center of gravity can result in deformation of the landing technique. Therefore, maintaining core muscle stability in athletes upon landing is crucial [10, 60, 61]. The success of this phase determines the quality of the entire technical action. Aerials athletes must have strong core muscle groups, a typical feature of athletes’ physical quality.

**Fig 7:**
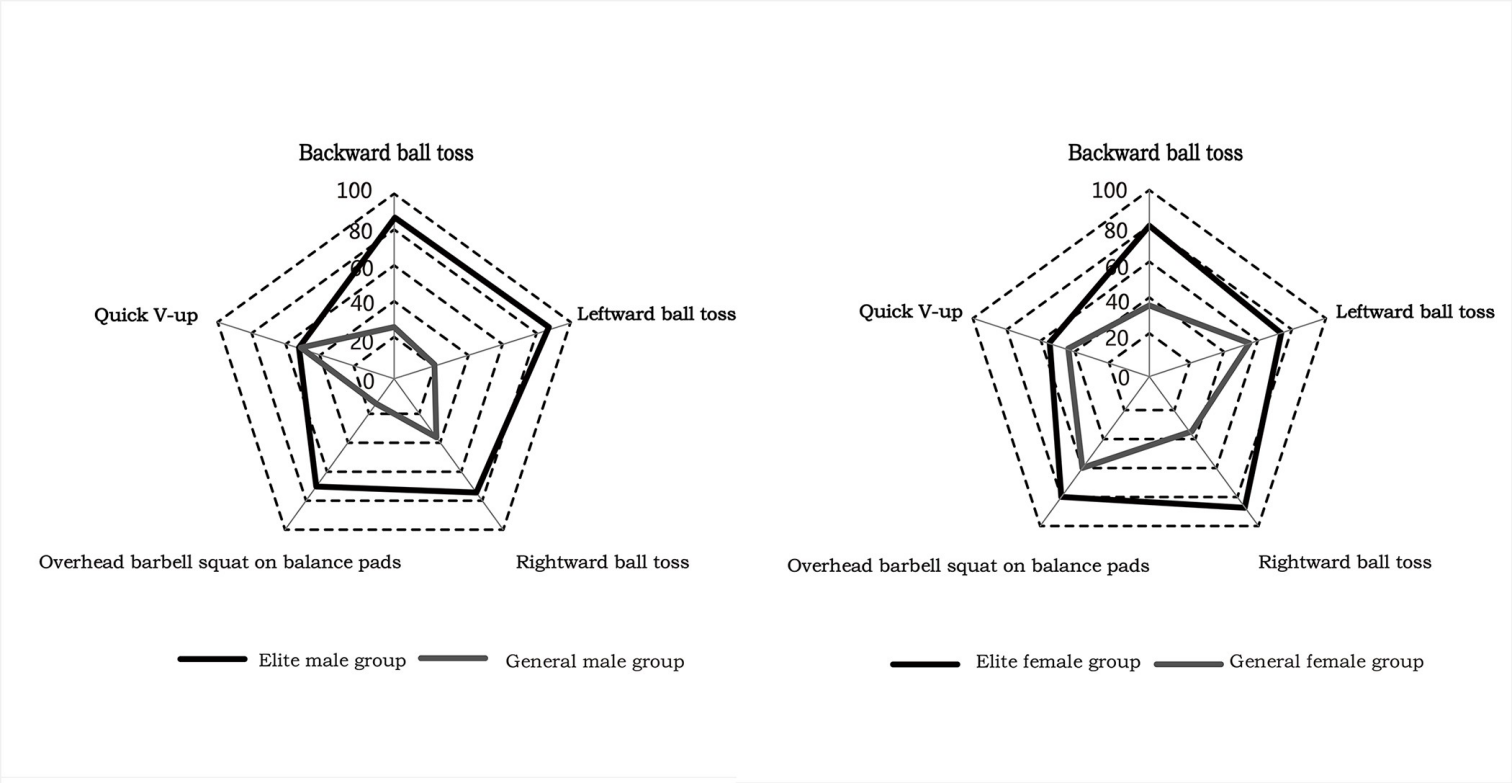
Comparison of specialized core strength test results in different groups.

Due to the high requirements of aerials on the core muscles, this study reflected the athletes’ specific explosive core strength and core stability strength index through graph processing. The differences between the groups were analyzed by visualizing the core-specific strength index data of each group for comparison. Because the numerical units of each index are different, the values of each index were first converted into percentiles and then plotted. The specific steps are as follows: (1) Determining the minimum and maximum values for each index, followed by calculating the scores at key percentiles, starting from the 1st, 2nd, 3rd, and extending through to the 99th and 100th; (2) The corresponding percentiles of the mean values of the five indexes were determined; (3) The radar chart for the comparison of the unique core strength test results of different groups of athletes was drawn up (a smaller numerical value for overhead barbell squat on balance pads and quick v-up indicated a low optimal index, while greater numerical values for the other indexes indicated a high optimal index). Fig 7 shows that each elite group’s unique core strength values were within the closed pentagon’s outer layer (radar chart). This indicated that the elite group had better unique explosive core strength and core stability strength. This also lays a solid foundation for elite athletes to perform more difficult aerial unique techniques and stabilize their ability to land.

**Speed characteristics.** The aerials event of freestyle skiing is technically characterized by its brief duration. From the perspective of energy metabolism, the movement takes less than 10 seconds, and the aerobic oxidation energy supply system is the main contributor for the sliding phase. The athletes also bear low load intensity. The key to determining the outcome of the competition is the aerial maneuver and landing phases, a period of rapid exertion of athletes lasting for 2–3 second. In this short technical movement, the body’s energy sources mainly rely on the ATP-CP energy supply system [9]. Aerial maneuver and landing require athletes to have good quick response ability. Executing technical maneuvers with quickness, precision, and stability is crucial for achieving high-quality performance. Consequently, coaches should focus significantly on enhancing both the speed and quality of training, aiming to improve athletes’ overall performance level. The 30-meter sprint effectively evaluates the ATP-CP energy supply system and serves as a reliable index of athletes’ speed quality. It reflects the body’s overall movement, the coordinated operation ability of muscles, and the agility of human hands and feet. Figs 5 and 6 show that the 30-meter sprint test results of the elite athletes were significantly better than those of the general athletes (P<0.05). This reflects that elite athletes have faster muscle contraction and reaction ability.

#### Endurance characteristics

Freestyle skiing aerials is a sport based on aerobic ability, although anaerobic ability plays a decisive role. The time interval for each athlete’s complete movement exceeds 5 minutes, with the average duration of competition or training sessions ranging between 120 to 240 minutes [9]. The waiting period during competition or training mainly depends on the aerobic oxidation energy supply system to promote the energy recovery of the ATP-CP system. Considering the energy supply characteristics of aerials, the unique physical fitness of aerials athletes should include aerobic endurance. Aerobic endurance is the body’s capacity to sustain a certain intensity load for a prolonged period, underpinned by adequate oxygen supply. Figs 5 and 6 illustrate that the 12-minute run test revealed no significant difference between the elite and general groups (P>0.05). This aligns with the aforementioned research findings on aerobic metabolic capacity.

#### Agility characteristics

Agile running primarily evaluates an athlete’s agility and coordination, signifying body sensitivity via straight sprints and directional changes. This reflects an athlete’s speed, adaptability, and rapid maneuvering abilities [58]. For freestyle skiing aerials athletes, the capacity to control limbs and maintain body balance on slippery snow is fundamental to technical movements. Coordinated footwork aids in adjusting takeoff distances, preparing for swift launch, and achieving high-speed performance in competitions [3, 62, 63].

As the technical complexity of freestyle skiing aerials evolves, athletes face increasing demands on movement difficulty and quality, necessitating enhanced limb and trunk coordination [5, 6]. The intricate and variable structure of aerials requires athletes to have quick, coordinated movements and precise muscular strength. Hence, agility becomes a vital asset in this sport, playing a critical role in overall technical movements. As Figs 5 and 6, significant differences were noted in men’s and women’s agility running results across different groups (P<0.01 for men; P<0.05 for women), suggesting distinct agility levels among freestyle skiing aerials athletes of different ranks.

### Analysis of factors contributing to physical fitness differences among different groups

Age and years of training significantly impact athletes’ competitive abilities. These two factors are crucial for the selection of prospective sports talents, training quality, and achieving excellent competition results. According to feedback from interviews with coaches, China’s national freestyle skiing aerials team is currently undergoing a transition phase between veteran and new athletes following the Beijing Winter Olympics. Due to the impact of the COVID-19 pandemic and other factors, there is a significant gap in competitive capabilities between reserve talents and current top athletes. An analysis of age and years of training (see Table 1) shows that athletes in the general group in China are generally younger and have fewer years of training, compared to the elite group. This difference reveals that, whether in terms of technical proficiency or physical reserves, athletes in the general group have substantial room for development. Elite athletes have undergone long-term systematic and cyclical training. This training regimen not only promotes their comprehensive development in technical skills but also significantly shapes their unique physical fitness characteristics.

### Limitations

This study has certain limitations. First, the small sample size may limit the generalizability of the results, which is a common issue in research involving elite athletes. Second, the lack of international athlete data prevents cross-national comparisons; future studies should include a broader sample. Additionally, due to the variability inherent in physical fitness testing, the data collected may not fully reflect the athletes’ optimal condition. Lastly, balance ability is also crucial for freestyle skiing aerials athletes. Although this ability was indirectly assessed through overhead barbell squat on balance pads and three-step stable jump, the study design and testing conditions did not allow for an independent and detailed analysis of balance. Future research should employ more precise testing methods to comprehensively evaluate athletes’ balance abilities under varied postural controls.

## Conclusion

This study has summarized the physical fitness characteristics of elite freestyle skiing aerials athletes. Compared to general athletes, elite athletes exhibit higher fat-free body weight, larger waist and thigh circumferences in terms of body morphology. Particularly, male athletes exhibit significantly lower body fat percentages. In terms of physiological function, these athletes demonstrate a stronger anaerobic metabolic capacity, which is crucial for high-intensity performance over short durations. In terms of physical qualities, they have more prominent limb strength, lower limb explosive power, and specialized core strength. They also display higher levels of speed and agility, and overall movement coordination. The superior physical performance of elite athletes can be attributed to variations in age, longer training years, and the structured and periodic scheduling of long-term training programs.

## Practical applications

Analyzing the physical fitness characteristics of elite athletes helps practitioners to scientifically diagnose and rationally plan training processes, thereby improving the quality and effectiveness of training. In terms of body morphology, aerials athletes need to focus on increasing muscle mass while maintaining a low body fat percentage and ensuring that body weight remains within the ideal range. Although the elite group has larger waist and thigh circumferences than the general group, training should not solely focus on increasing body dimensions but rather emphasize strength development. The body morphology characteristics of elite athletes are the result of years of systematic and cyclical training, reflecting the cumulative effects of training intensity and volume. In terms of physiological function, aerials athletes should prioritize the development of anaerobic capacity while also enhancing aerobic capacity, to meet the energy metabolism demands of the aerials event. In terms of physical qualities, coaches must ensure that athletes enhance limb strength and lower limb explosive power while maintaining symmetry in muscle strength. Targeted core strength training should be designed based on technical characteristics to strengthen athletes’ core muscles, thereby ensuring precise execution of technical maneuvers. Additionally, other physical qualities such as speed and agility should be systematically enhanced to optimize athletes’ overall athletic performance and neuromuscular coordination.

## Supporting information

S1 Appendix Expert interview outline. (PDF)

S2 Appendix Index survey questionnaire and results. (PDF)

S3 Appendix Questionnaire validity assessment and results. (PDF)

S4 Appendix Detailed rules for testing. (PDF)

S5 Appendix Statistical analysis results. (PDF)

## References

[1] Ge B. Study on aerials of freestyle skiing. Beijing: The People’s Sports Publishing House; 2003. pp. 7–8.

[2] Yeadon MR. The limits of aerial twisting techniques in the aerials event of freestyle skiing. J Biomech. 2013;46(5):1008–13. doi: 10.1016/j.jbiomech.2012.11.029 23235111

[3] Jiang D, Wang H, Chen J, Dong C. Precise prediction of launch speed for athletes in the aerials event of freestyle skiing based on deep transfer learning. Sci Rep. 2023;13(1). doi: 10.1038/s41598-023-31355-8 36922628 PMC10017692

[4] Winter Sports Management Center of China. Freestyle skiing competition rules and judges’ handbook. Beijing: People’s Sports Publishing House; 2010.

[5] Qiu S, Shi D, Liu L, Ge B, Qiu Z. Research on the gold-medal winning strategy for Chinese women’s freestyle skiing aerials in the Beijing 2022 Olympic Winter Games. J Beijing Sport Univ. 2021;44(12). doi: 10.19582/j.cnki.11-3785/g8.2021.12.006

[6] Li H, Zhang LC, Wang JR, Liu J, Sun YL. Executive control of freestyle skiing aerials athletes in different training conditions. Front Psychol. 2022;13: 12. doi: 10.3389/fpsyg.2022.968651 36225691 PMC9549268

[7] Yeadon MR, Hiley MJ. The control of twisting somersaults. J Biomech. 2014;47(6):1340–7. doi: 10.1016/j.jbiomech.2014.02.006 24576588

[8] Yeadon MR. Twisting techniques used in freestyle aerial skiing. Int J Sport Biomech. 1989;5(2):275–81. doi: 10.1123/ijsb.5.2.275

[9] Xuesong N. Research on physical fitness training for freestyle skiing aerials. Beijing: Beijing Sport University Publishing House; 2016.

[10] Wei M, Fan Y, Ren H, Li K, Niu X. Correlation between core stability and the landing kinetics of elite aerial skiing athletes. Sci Rep. 2023;13. doi: 10.1038/s41598-023-38435-9 37433875 PMC10336043

[11] Lou Y, Hao W, Fan W, Li Y, Wu C. Biomechanical research progress on landing stability of freestyle skiing aerials athletes. Chin J Sports Med. 2021;40(03):237–44. doi: 10.16038/j.1000-6710.2021.03.014

[12] Fu XL, Du L, Song YP, Chen HL, Shen WQ. Incidence of injuries in professional snow sports: A systematic review and meta-analysis. J Sport Health Sci. 2022;11(1):6–13. doi: 10.1016/j.jshs.2020.10.006 33130094 PMC8847944

[13] Ming W, Xuesong N, Nan L. Application and analysis of rehabilitation training after anterior cruciate ligament reconstruction surgery for Xu Mengtao. China Sport Sci. 2021;41(08):25–33. doi: 10.16469/j.css.202108004

[14] Wu Y, Dai RL, Yan WQ, Ren S, Ao YF. Characteristics of sports injuries in athletes during the Winter Olympics: A systematic review and meta-analysis. Orthop J Sports Med. 2023;11(12):9. doi: 10.1177/23259671231209286 38107844 PMC10722932

[15] Nayun C, Yingfang A, Yanfang J, Xiaoqing H. Characteristics of severe knee joint injuries in elite freestyle skiing aerials athletes: A study based on 11 members of the Chinese national team. Chin J Sports Med. 2019;38(7):543–7. doi: 10.16038/j.1000-6710.2019.07.001

[16] Yi M, Hongguang Y, Kai Z, Yantao L. Research on the relations between take-off technology, lower extremities power and landing stability for freestyle skiing aerials athletes. Chin Sport Sci Technol. 2012;48(03):64–8. doi: 10.16470/j.csst.2012.03.014

[17] Fu Y, Wang X, Yu T. Simulation analysis of knee ligaments in the landing phase of freestyle skiing aerial. Appl Sci. 2019;9(18):3713. doi: 10.3390/app9183713

[18] Yao Y, Niu X. Construction of a physical fitness evaluation index system and model for high-level freestyle skiing aerials athletes in China. PLoS One. 2023;18(12):e0295622. doi: 10.1371/journal.pone.0295622 38064528 PMC10707543

[19] Montgomery MM, Shultz SJ, Schmitz RJ, Wideman L, Henson RA. Influence of lean body mass and strength on landing energetics. Med Sci Sports Exerc. 2012;44(12):2376–83. doi: 10.1249/MSS.0b013e318268fb2d 22811034

[20] Wei M, Fan Y, Lu Z, Niu X, Wu H. Eight weeks of core stability training improves landing kinetics for freestyle skiing aerials athletes. Front Psychol. 2022;13: 994818. doi: 10.3389/fphys.2022.994818 36406981 PMC9669898

[21] Xuesong N, Ye B, Haiying R. Applied research of specific strength training of freestyle skiing aerials at the Sochi Winter Olympics. J Chengdu Sport Univ. 2015;41(05):111–6. doi: 10.15942/j.jcsu.2015.05.021

[22] Tomlin DL, Wenger HA. The relationship between aerobic fitness and recovery from high intensity intermittent exercise. Sports Med. 2001;31(1):1–11. doi: 10.2165/00007256-200131010-00001 11219498

[23] Mengtao X, Wei F, Zepeng L, Yantao L. Science and technology promoting female freestyle sking aerials athletes’preparation for the Beijing Winter Olympies circle. J Shenyang Sport Univ 2022;41(05):1–7. doi: 10.12163/j.ssu.20220704

[24] Alvarez-San Emeterio C, Gonzalez-Badillo JJ. The physical and anthropometric profiles of adolescent alpine skiers and their relationship with sporting rank. J Strength Cond Res. 2010;24(4):1007–12. doi: 10.1519/JSC.0b013e3181cbabb5 20300026

[25] Jones TW, Lindblom HP, Karlsson O, Andersson EP, McGawley K. Anthropometric, physiological, and performance developments in cross-country skiers. Med Sci Sports Exerc. 2021;53(12):2553–64. doi: 10.1249/MSS.0000000000002739 34649265

[26] Orvanova E. Physical structure of winter sports athletes. J Sports Sci. 1987;5(3):197–248. doi: 10.1080/02640418708729779 3330994

[27] Vernillo G, Pisoni C, Thiebat G. Physiological and physical profile of snowboarding: a preliminary review. Front Physiol. 2018;9: 7. doi: 10.3389/fphys.2018.00770 29973888 PMC6019472

[28] Polat M. An examination of respiratory and metabolic demands of alpine skiing. J Exerc Sci Fit. 2016;14(2):76–81. doi: 10.1016/j.jesf.2016.10.001 29541122 PMC5801720

[29] Schobersberger W, Mairhofer M, Haslinger S, Koller A, Raschner C, Puntscher S, et al. Are there associations between submaximal and maximal aerobic power and international ski federation world cup ranking in elite alpine skiers? Int J Sport Physiol Perform. 2021;16(5):628–33. doi: 10.1123/ijspp.2020-0105 33508781

[30] Turnbull JR, Kilding AE, Keogh JWL. Physiology of alpine skiing. Scand J Med Sci Sports. 2009;19(2):146–55. doi: 10.1111/j.1600-0838.2009.00901.x 19335589

[31] White AT, Johnson SC. Physiological comparison of international, national and regional alpine skiers. Int J Sports Med. 1991;12(4):374–8. doi: 10.1055/s-2007-1024697 1917221

[32] Fasel B, Sporri J, Gilgien M, Boffi G, Chardonnens J, Muller E, et al. Three-dimensional body and centre of mass kinematics in alpine ski racing using differential GNSS and inertial sensors. Remote Sens. 2016;8(8):13. doi: 10.3390/rs8080671

[33] Hintermeister RA, Oconnor DD, Dillman CJ, Suplizio CL, Lange GW, Steadman JR. Muscle activity in slalom and giant slalom skiing. Med Sci Sports Exerc. 1995;27(3):315–22. doi: 10.1249/00005768-199503000-00005 7752856

[34] Muller L, Muller E, Kornexl E, Raschner C. The relationship between physical motor skills, gender and relative age effects in young austrian alpine ski racers. Int J Sports Sci Coach. 2015;10(1):69–85. doi: 10.1260/1747-9541.10.1.69

[35] Platzer HP, Raschner C, Patterson C, Lembert S. Comparison of physical characteristics and performance among elite snowboarders. J Strength Cond Res. 2009;23(5):1427–32. doi: 10.1519/JSC.0b013e3181aa1d9f 19620923

[36] Kroshus E, Wagner J, Wyrick D, Athey A, Bell L, Benjamin HJ, et al. Wake up call for collegiate athlete sleep: narrative review and consensus recommendations from the NCAA Interassociation Task Force on Sleep and Wellness. Br J Sports Med. 2019;53(12):731–6. doi: 10.1136/bjsports-2019-100590 31097460

[37] Rodriguez-Manas L, Feart C, Mann G, Vina J, Chatterji S, Chodzko-Zajko W, et al. Searching for an operational definition of frailty: a Delphi method based consensus statement: the frailty operative definition-consensus conference project. J Gerontol A Biol Sci Med Sci. 2013;68(1):62–7. doi: 10.1093/gerona/gls119 22511289 PMC3598366

[38] Weir A, Holmich P, Schache AG, Delahunt E, de Vos RJ. Terminology and definitions on groin pain in athletes: building agreement using a short Delphi method. Br J Sports Med. 2015;49(12):825–7. doi: 10.1136/bjsports-2015-094807 25907180 PMC4484360

[39] Yuan J, Huang H. Sports measurement and evaluation. Beijing: The People’s Sports Publishing House; 2011. pp. 61–78.

[40] Nakagawa S, Cuthill IC. Effect size, confidence interval and statistical significance: a practical guide for biologists. (vol 82, p. 591, 2007). Biol Rev. 2009;84(3):515. doi: 10.1111/j.1469-185X.2009.00083.x 17944619

[41] Maher JM, Markey JC, Ebert-May D. The other half of the story: Effect size analysis in quantitative research. CBE Life Sci Educ. 2013;12(3):345–51. doi: 10.1187/cbe.13-04-0082 24006382 PMC3763001

[42] Bayios IA, Bergeles NK, Apostolidis NG, Noutsos KS, Koskolou MD. Anthropometric, body composition and somatotype differences of Greek elite female basketball, volleyball and handball players. J Sports Med Phys Fitness. 2006;46(2):271–80. doi: 10.1016/j.jsams.2006.03.024 16823358

[43] Gabbett T, Georgieff BJTJoS, Research C. Physiological and anthropometric characteristics of Australian junior national, state, and novice volleyball players. 2007;21(3):902–8. doi: 10.1519/R-20616.1 17685708

[44] Stanula A, Roczniok R, Gabrys T, Szmatlan-Gabrys U, Maszczyk A, Pietraszewski P. Relations between BMI, body mass and height, and sports competence among participants of the 2010 Winter Olympic Games: does sport metabolic demand differentiate? Percept Mot Skills. 2013;117(3):837–54. doi: 10.2466/25.29.PMS.117x31z4 24665801

[45] Stoggl T, Enqvist J, Muller E, Holmberg HC. Relationships between body composition, body dimensions, and peak speed in cross-country sprint skiing. J Sports Sci. 2010;28(2):161–9. doi: 10.1080/02640410903414160 20391090

[46] Xu Y, Yang C, Yang Y, Zhang X, Zhang S, Zhang M, et al. A narrative review of injury incidence, location, and injury factor of elite athletes in snowsport events. Front Psychol. 2020;11:589983. doi: 10.3389/fphys.2020.589983 33488394 PMC7820716

[47] Dewig DR, Goodwin JS, Pietrosimone BG, Blackburn JT. Associations among eccentric hamstrings strength, hamstrings stiffness, and jump-landing biomechanics. J Athl Train. 2020;55(7):717–23. doi: 10.4085/1062-6050-151-19 32432902 PMC7384474

[48] Neamatallah Z, Herrington L, Jones R. An investigation into the role of gluteal muscle strength and EMG activity in controlling HIP and knee motion during landing tasks. Phys Ther Sport. 2020;43:230–5. doi: 10.1016/j.ptsp.2019.12.008 31902735

[49] Bringhurst RF, Wagner DR, Schwartz S. Wingate anaerobic test reliability on the Velotron with ice hockey players. J Strength Cond Res. 2020;34(6):1716–22. doi: 10.1519/JSC.0000000000002458 29385006

[50] Collomp K, Ahmaidi S, Audran M, Chanal JL, Prefaut C. Effects of caffeine ingestion on performance and anaerobic metabolism during the Wingate test. Int J Sports Med. 1991;12(5):439–43. doi: 10.1055/s-2007-1024710 1752708

[51] Rabadan M, Diaz V, Calderon FJ, Benito PJ, Peinado AB, Maffulli N. Physiological determinants of speciality of elite middle- and long-distance runners. J Sports Sci. 2011;29(9):975–82. doi: 10.1080/02640414.2011.571271 21604227

[52] Melo X, Arrais I, Maroco JL, Ribeiro PN, Nabais S, Coelho R, et al. Effects of kettlebell swing training on cardiorespiratory and metabolic demand to a simulated competition in young female artistic gymnasts. PLoS One. 2023;18(4):17. doi: 10.1371/journal.pone.0283228 37093847 PMC10124852

[53] Xuesong N, Yi M. Applied research on freestyle skiing aerials physical training monitoring of China. J Shenyang Sport Univ 2011;30(04):15–9. doi: 10.3969/j.issn.1004-0560.2011.04.004

[54] Maijiu T. Event-group training theory. Beijing: The People’s Sports Publishing House; 1998.

[55] Feng L. Method for evaluating the physiological function of excellent athletes. Beijing: The People’s Sports Publishing House; 2003.

[56] Suchomel TJ, Nimphius S, Stone MH. The Importance of muscular strength in athletic performance. Sports Med. 2016;46(10):1419–49. doi: 10.1007/s40279-016-0486-0 26838985

[57] James LP, Suchomel TJ, Comfort P, Haff GG, Connick MJ. Rate of force development adaptations after weightlifting-style training: the influence of power clean ability. J Strength Cond Res. 2022;36(6):1560–7. doi: 10.1519/JSC.0000000000003673 35622107

[58] Wang R. Exercise physiology. Beijing: The People’s Sports Publishing House; 2012.

[59] Hauw D, Renault G, Durand M. How do aerial freestyler skiers land on their feet? A situated analysis of athletes’ activity related to new forms of acrobatic performance. J Sci Med Sport. 2008;11(5):481–6. doi: 10.1016/j.jsams.2007.06.005 17706460

[60] Kibler WB, Press J, Sciascia A. The role of core stability in athletic function. Sports Med. 2006;36(3):189–98. doi: 10.2165/00007256-200636030-00001 16526831

[61] Okada T, Huxel KC, Nesser TW. Relationship between core stability, functional movement, and performance. J Strength Cond Res. 2011;25(1):252–61. doi: 10.1519/JSC.0b013e3181b22b3e 20179652

[62] Sheppard JM, Young WB. Agility literature review: Classifications, training and testing. J Sports Sci. 2006;24(9):919–32. doi: 10.1080/02640410500457109 16882626

[63] Liu Z, Li D, Fu Y, Xin W. The effects of changing site size on the athletes’ take-off speed in freestyle skiing aerial skills. Chin Sport Sci Technol. 2020;56(12):78–82. doi: 10.16470/j.csst.2020095

