# Supplementary material for: Physical fitness characteristics of elite freestyle skiing aerials athletes
Source: PLoS One. 2024 Jun 6;19(6):e0304912. doi: 10.1371/journal.pone.0304912 (PMC11156283; doi:10.1371/journal.pone.0304912)
Supplement: S1 Appendix — (PDF) [file pone.0304912.s001.pdf]

Appendix 1

Expert Interview Outline

Expert (Coach) Name:________________

Expert (Coach) Title： ________________

1.What abilities do you believe constitute the competitive capability of freestyle skiing aerials athletes?

2.How important do you think physical fitness is to freestyle skiing aerials athletes?

3.What do you believe are the essential elements of physical fitness in the freestyle skiing aerials discipline?

4.In your opinion, what are the key factors for success in freestyle skiing aerials?

5.What do you think are the characteristics of energy metabolism in the freestyle skiing aerials discipline?

6. What do you believe is the ideal body morphology for athletes in the freestyle skiing aerials discipline?

7.What do you believe are the essential physiological functions required for athletes in the freestyle skiing aerials discipline?

8.What kind of physical capacity do you believe athletes in the freestyle skiing aerials discipline should possess?

9. Which indexes do you consider as representative for evaluating body morphology?

10. Which indexes do you consider as representative for evaluating physiological function?

11.Which indexes do you consider as representative for evaluating physical capacity?
