# Supplementary material for: Physical fitness characteristics of elite freestyle skiing aerials athletes
Source: PLoS One. 2024 Jun 6;19(6):e0304912. doi: 10.1371/journal.pone.0304912 (PMC11156283; doi:10.1371/journal.pone.0304912)
Supplement: S2 Appendix — (PDF) [file pone.0304912.s002.pdf]

Appendix 2-1

Expert questionnaire on physical fitness index selection for elite freestyle skiing aerials athletes

Respected Expert,

Greetings!

Thank you immensely for taking the time out of your busy schedule to complete this questionnaire. Through this inquiry form, we aim to understand your views and opinions on the physical fitness evaluation indexes for China's elite freestyle skiing aerials athletes. The insights garnered will serve as a reference for the selection and training of athletes in this discipline in China.

To ensure the authenticity and effectiveness of this research, we kindly ask that you respond to the questions in the following table based on your true opinions and beliefs. Your contribution is deeply appreciated, and I would like to extend my sincere gratitude for your support and assistance. Please rest assured that your answers will be used strictly for this research, and we will maintain the confidentiality of your personal information.

Once again, I express my sincere gratitude for your invaluable assistance.

1.Your basic information

Name：	
Age:	
Gender:	
Affiliation:	
Position:	
Professional title:	
Educational Level:	
Research Area:	
Years of Teaching or Training Experience:	

2.Index scoring

For the indexes provided in the table below, please assign a score based on their relevance to specialized physical fitness. The scores are categorized into five levels: Very Important = 5 points, Quite Important = 4 points, Neutral = 3 points, Not Important = 2 points, Very Unimportant = 1 point. Kindly mark "√" under the corresponding score.

Table 1 First-level index system

First-level index	Agree	Disagree	Suggested modifications	Importance Level				
				5	4	3	2	1
Body morphology								
Physiological function								
Physical quality								

Table 2 Second-level index system

First-level index	Second-level index	Agree	Disagree	Suggested modifications	Importance Level				
					5	4	3	2	1
Body morphology	Body length								
	Body width								
	Body circumference								
	Body composition								
Physiological function	Cardiopulmonary performance								
	Aerobic ability								
	Oxygen usage ability								
	Energy metabolism and endocrine system								
Physical quality	Strength								
	Speed								
	Endurance								
	Sensitivity								
	Flexibility								

Table 3 Third-level index system

First-level index	Second-level index	Third-level index	Agree	Disagree	Suggested modifications	Importance Level				
						5	4	3	2	1
Body morphology	Length	Height								
		Sitting height								
		Upper limb length								
		Lower limb length								
		Lower leg length								
		Achilles tendon length								
	Width	Shoulder width								
		Hip width								
		Pelvis wide								
	Circumference	Chest circumference								
		Waist circumference								
		Hip circumference								
		Thigh circumference								
		Calf circumference								
		Ankle circumference								
	Body composition	Body fat percentage								
		Body fat percentage								
		Fat-free body weight								
		Waist-hip ratio								
		Body mass index								
		Quetelet index								
		Verwaeck index								
Physiological function	Cardiopulmonary function	Maximal oxygen intake								
		Relative maximum oxygen uptake								
		Resting heart rate								
		Maximum heart rate								
	Anaerobic capacity	Maximum anaerobic power								
		Relative maximum anaerobic power								

		Average anaerobic power								
		Relative average anaerobic power								
	Oxygen usage ability	Hemoglobin								
		Red blood cell count								
		Hematocrit								
	Energy metabolism and endocrine system	Blood urea								
		Serum creatine kinase								
		Blood lactate								
		Serum testosterone								
		Serum cortisol								
Physical quality	Strength	Bench press								
		Barbell squat								
		Pull up								
		Power clean								
		Side throw (left)								
		Side throw (right)								
		Back throw								
		Single-leg triple jump (left)								
		Single-leg triple jump (right)								
		Jump deep high								
		Standing long jump								
		Overhead barbell squat on balance pads								
		Nordic hamstring curl								
		Quick v-up								
		Leg raise on parallel bars								
	Speed	30-meter sprint								
		100-Meter Sprint								
	Endurance	12-minute run								
		40-Minute Run								
		3000-Meter Run								

	Agility	Agile running								
		Cross-directional jump								
		Hexagonal jump								
	Flexibility	Seated forward bend								
		Splits (left and right leg front splits and middle split)								

The above are questions designed specifically for this research. Due to our limited knowledge, if you see any shortcomings or areas of improvement, please leave your valuable feedback:

____

____

____

Once again, we sincerely thank you for your strong support!

3.Expert familiarity and judgement basis

Table 4: Your familiarity with the Indexes

**Familiar（1.0）**	**Somewhat Familiar（0.8）**	**Neutral（0.5）**	**Not Very Familiar（0.2）**	**Unfamiliar（0.0）**

Table 5: Your basis for judging the indexes

Judgment basis	Level of judgment basis		
	High	Medium	Low
Practical experience	0.5	0.4	0.3
Theoretical analysis	0.3	0.2	0.1
Referenced literature	0.1	0.1	0.1
Personal intuition	0.1	0.1	0.1

Appendix 2-2

1. Expert questionnaire on physical fitness index selection for elite freestyle skiing aerials athletes (First round)

Table 1 First-level index system

**Number**	**First-level index**	**Agreement rate**	**Suggested modifications**	**M±SD**	**Coefficient of Variation**	**Result**
A1	Body morphology	100%		4.42±0.49	0.11	Retention
A2	Physiological function	100%		4.58±0.64	0.14	Retention
A3	Physical quality	100%		4.83±0.37	0.08	Retention

Table 2 Second-level index system

**Number**	Second-level index	**Agreement rate**	**Suggested modifications**	**M±SD**	**Coefficient of Variation**	**Result**
B1	Body length	100%		4.25±0.60	0.14	Retention
B2	Body width	100%		4.33±0.47	0.11	Retention
B3	Body circumference	100%		4.75±0.43	0.09	Retention
B4	Body composition	100%		4.33±0.47	0.11	Retention
B5	Cardiopulmonary performance	100%		4.42±0.76	0.17	Retention
B6	Aerobic ability	100%		4.75±0.43	0.09	Retention
B7	Oxygen usage ability	100%		4.42±0.64	0.14	Retention
B8	Energy metabolism and endocrine system	100%		4.58±0.49	0.11	Retention
B9	Strength	100%		4.83±0.37	0.08	Retention
B10	Speed	100%		4.25±0.43	0.10	Retention
B11	Endurance	100%		4.33±0.62	0.14	Retention
B12	Agility	100%		4.50±0.50	0.11	Retention
B13	Flexibility	58.33%	Relevance to the study is relatively low.	3.33±0.75	0.22	Deletion

Table 3. Third-level index system

**Number**	Third-level index	**Agreement rate**	**Suggested modifications**	**M±SD**	**Coefficient of Variation**	**Result**
C1	Height	100%		4.33±0.62	0.14	Retention
C2	Sitting height	100%	Similar to the height index	3.08±0.64	0.21	Deletion
C3	Upper limb length	100%		4.25±0.72	0.17	Retention
C4	Lower limb length	100%		4.50±0.50	0.11	Retention
C5	Lower leg length	66.67%	The calf length requirement is not high	3.00±0.71	0.24	Deletion
C6	Achilles tendon length	100%		4.42±0.64	0.14	Retention
C7	Shoulder width	100%		4.25±0.60	0.14	Retention
C8	Hip width	41.67%	Adopting pelvis wide	3.33±0.62	0.19	Deletion
C9	Pelvis wide	100%		4.42±0.76	0.17	Retention
C10	Chest circumference	66.67%	Low requirements for chest circumference	3.17±0.55	0.17	Deletion
C11	Waist circumference	100%		4.67±0.47	0.10	Retention
C12	Hip circumference	100%		4.08±0.64	0.16	Retention
C13	Thigh circumference	100%		4.67±0.62	0.13	Retention
C14	Calf circumference	100%		4.25±0.60	0.14	Retention
C15	Ankle circumference	66.67%	Not prominently related to the specialization	3.17±0.55	0.17	Deletion
C16	Body fat percentage,	100%		4.33±0.75	0.17	Retention
C17	Body fat percentage,	100%		4.50±0.50	0.11	Retention
C18	Fat-free body weight	100%		4.67±0.47	0.10	Retention
C19	Waist-hip ratio	100%		4.17±0.55	0.13	Retention
C20	Body mass index	100%		4.25±0.60	0.14	Retention
C21	Quetelet index	100%		4.42±0.76	0.17	Retention
C22	Verwaeck index	41.67%	Not prominently related to the specialization	3.08±0.64	0.21	Deletion
C23	Maximal oxygen intake	100%		4.50±0.65	0.14	Retention

C24	Relative maximum oxygen uptake	100%		4.67±0.67	0.10	Retention
C25	Resting heart rate	33.33%	Not closely related to the specialization	2.75±0.60	0.22	Deletion
C26	Maximum heart rate	58.33%	Adopting maximum anaerobic power	3.58±0.64	0.18	Deletion
C27	Maximum anaerobic power	100%		4.67±0.47	0.10	Retention
C28	Relative maximum anaerobic power	100%		4.75±0.43	0.09	Retention
C29	Average anaerobic power	58.33%	Adopting maximum anaerobic power	3.33±0.47	0.14	Deletion
C30	Relative average anaerobic power	66.67%	Adopting relative maximum anaerobic power	3.25±0.72	0.22	Deletion
C31	Hemoglobin	100%		4.25±0.60	0.14	Retention
C32	Red blood cell count	100%		4.17±0.55	0.13	Retention
C33	Hematocrit	50%	Adopting red blood cell count	3.42±0.76	0.22	Deletion
C34	Blood urea	100%		4.42±0.64	0.14	Retention
C35	Serum creatine kinase	100%		3.67±0.47	0.13	Deletion
C36	Blood lactate	100%	Not closely related to the specialization	3.00±0.58	0.19	Deletion
C37	Serum testosterone	100%		4.58±0.64	0.14	Retention
C38	Serum cortisol	100%		4.42±0.49	0.11	Retention
C39	Bench press	100%		4.08±0.86	0.21	Retention
C40	Barbell squat	100%		4.83±0.37	0.08	Retention
C41	Pull up	100%		4.67±0.47	0.10	Retention
C42	Power clean	100%		4.75±0.43	0.09	Retention
C43	Side throw (left)	100%		4.83±0.37	0.08	Retention
C44	Side throw (right)	100%		4.67±0.67	0.10	Retention
C45	Back throw	100%		4.58±0.51	0.11	Retention
C46	Single-leg triple jump (left)	100%		4.75±0.60	0.13	Retention
C47	Single-leg triple jump (right)	100%		4.75±0.60	0.13	Retention

C48	Jump deep high	58.33%	Adopting standing long jump	3.00±0.58	0.19	Deletion
C49	Standing long jump	100%		4.42±0.49	0.11	Retention
C50	Overhead barbell squat on balance pads	100%		4.58±0.64	0.14	Retention
C51	Nordic hamstring curl	50%	The difficulty level of the index is too high	3.25±0.43	0.13	Deletion
C52	Quick v-up	100%		4.50±0.65	0.14	Retention
C53	Leg raise on parallel bars	33.33%	Adopting quick v-up	3.42±0.76	0.22	Deletion
C54	30-meter sprint	100%	Pay attention to the testing details	4.25±0.43	0.10	Retention
C55	100-Meter Sprint	25%	Using the 30-meter sprint is more in line with the specific energy demands of the discipline	3.33±0.62	0.19	Deletion
C56	12-minute run	100%		4.25±0.60	0.14	Retention
C57	40-minute Run	100%		3.08±0.64	0.21	Deletion
C58	3000-meter Run	100%	Adopting 12-minute run	3.42±0.49	0.14	Deletion
C59	Agile running	100%		4.42±0.49	0.11	Retention
C60	Cross-directional jump	100%	Adopting agile running	3.08±0.49	0.16	Deletion
C61	Hexagonal jump	100%	Adopting agile running	3.00±0.58	0.19	Deletion
C62	Seated forward bend	33.33%	Relevance to the study is relatively low	3.50±0.50	0.14	Deletion
C63	Splits (left and right leg front splits and middle split)	25%	Relevance to the study is relatively low	3.67±0.47	0.13	Deletion

2. Summary of experts' familiarity and judgment basis results (First round)

Table 4: Survey results on experts' familiarity with the indexes (n=12)

**Degree of familiarity**	**Familiar（1.0）**	**Somewhat Familiar（0.8）**	**Neutral（0.5）**	**Not Very Familiar（0.2）**	**Unfamiliar（0.0）**
Number	6	4	2		

Table 5: Survey results on experts' judgment basis for the indexes (n=12)

Judgment basis	Level of judgment basis		
	High	Medium	Low
Practical experience	7	5	0
Theoretical analysis	4	6	2
Referenced literature	7	5	0
Personal intuition	8	4	0

Appendix 2-3

1. Expert questionnaire on physical fitness index selection for elite freestyle skiing aerials athletes (Second round)

Table 1 First-level index system

Number	First-level index	Agreement rate	Suggested modifications	M±SD	Coefficient of Variation	Result
A1	Body morphology	100%		4.58±0.49	0.11	Retention
A2	Physiological function	100%		4.75±0.43	0.09	Retention
A3	Physical quality	100%		4.92±0.28	0.06	Retention

Table 2 Second-level index system

Number	Second-level index	Agreement rate	Suggested modifications	M±SD	Coefficient of Variation	Result
B1	Body length	100%		4.25±0.60	0.14	Retention
B2	Body width	100%		4.42±0.49	0.11	Retention
B3	Body circumference	100%		4.50±0.50	0.11	Retention
B4	Body composition	100%		4.67±0.47	0.10	Retention
B5	Cardiopulmonary performance	100%		4.50±0.65	0.14	Retention
B6	Aerobic ability	100%		4.92±0.28	0.06	Retention
B7	Oxygen usage ability	100%		4.67±0.62	0.13	Retention
B8	Energy metabolism and endocrine system	100%		4.75±0.43	0.09	Retention
B10	Strength	100%		4.92±0.28	0.06	Retention
B11	Speed	100%		4.42±0.49	0.11	Retention
B12	Endurance	100%		4.33±0.75	0.17	Retention
B13	Agility	100%		4.58±0.49	0.11	Retention

Table 3 Third-level index system

Number	Third-level Index	Agreement rate	Suggested modifications	M±SD	Coefficient of Variation	Result
C1	Height	100%		4.17±0.55	0.13	Retention
C2	Upper limb length	100%		4.25±0.60	0.14	Retention
C3	Lower limb length	100%		4.33±0.47	0.11	Retention
C4	Achilles tendon length	100%		4.67±0.47	0.10	Retention
C5	Shoulder width	100%		4.50±0.65	0.14	Retention
C6	Pelvis wide	100%		4.58±0.64	0.14	Retention
C7	Waist circumference	100%		4.83±0.37	0.08	Retention
C8	Hip circumference	100%	Adopting waist-hip ratio	3.58±0.64	0.18	Deletion
C9	Thigh circumference	100%		4.83±0.37	0.08	Retention
C10	Calf circumference	100%		4.33±0.47	0.11	Retention
C11	Body weight	100%		4.50±0.65	0.14	Retention
C12	Body fat percentage	100%		4.75±0.43	0.09	Retention
C13	Fat-free body weight	100%		4.67±0.47	0.10	Retention
C14	Waist-hip ratio	100%		4.42±0.49	0.11	Retention
C15	Body mass index	100%		4.58±0.64	0.14	Retention
C16	Quetelet index	100%		4.67±0.47	0.10	Retention
C17	Maximal oxygen intake	100%		4.58±0.64	0.14	Retention
C18	Relative maximum oxygen uptake	100%		4.75±0.43	0.09	Retention
C19	Maximum anaerobic power	100%		4.83±0.37	0.08	Retention
C20	Relative maximum anaerobic power	100%		4.92±0.28	0.06	Retention
C21	Hemoglobin	100%		4.50±0.50	0.11	Retention
C22	Red blood cell count	100%		4.42±0.49	0.11	Retention
C23	Blood urea	100%		4.67±0.47	0.10	Retention
C24	Serum testosterone	100%		4.75±0.43	0.09	Retention
C25	Serum cortisol	100%		4.58±0.49	0.11	Retention
C26	Bench press	100%	Not closely related to the specialization	3.58±0.49	0.14	Deletion
C27	Barbell squat	100%		4.92±0.28	0.06	Retention

C28	Pull up	100%		4.75±0.43	0.09	Retention
C29	Power clean	100%		4.92±0.28	0.06	Retention
C30	Side throw (left)	100%		4.83±0.37	0.08	Retention
C31	Side throw (right)	100%		4.75±0.43	0.09	Retention
C32	Back throw	100%		4.75±0.43	0.09	Retention
C33	Single-leg triple jump (left)	100%		4.92±0.28	0.09	Retention
C34	Single-leg triple jump (right)	100%		4.92±0.28	0.09	Retention
C35	Standing long jump	100%		4.58±0.47	0.11	Retention
C36	Overhead barbell squat on balance pads	100%	Pay attention to the measurement method	4.83±0.37	0.08	Retention
C37	Quick v-up	100%		4.75±0.43	0.09	Retention
C38	30-meter sprint	100%		4.50±0.50	0.11	Retention
C39	12-minute run	100%		4.58±0.49	0.11	Retention
C40	Agile running	100%		4.67±0.47	0.10	Retention

2.Summary of experts' familiarity and judgment basis results (Second round)

Table 4 Survey results on experts' familiarity with the indexes (n=12)

Degree of familiarity	Familiar（1.0）	Somewhat Familiar（0.8）	Neutral（0.5）	Not Very Familiar（0.2）	Unfamiliar（0.0）
Number	**7**	**3**	**2**		

Table 5 Survey results on experts' judgment basis for the indexes(n=12)

Judgment basis	Level of judgment basis		
	High	Medium	Low
Practical experience	7	5	0
Theoretical analysis	8	3	1
Referenced literature	6	6	0
Personal intuition	9	3	0

Table 6 Summary of Expert Authority Level

Number of questionnaire surveys	Judgment basis	Degree of familiarity	Authority Coefficient
First Round	0.88	0.85	**0.86**
Second Round	0.92	0.87	**0.89**
