# Supplementary material for: Physical fitness characteristics of elite freestyle skiing aerials athletes
Source: PLoS One. 2024 Jun 6;19(6):e0304912. doi: 10.1371/journal.pone.0304912 (PMC11156283; doi:10.1371/journal.pone.0304912)
Supplement: S3 Appendix — (PDF) [file pone.0304912.s003.pdf]

Appendix 3

Expert validity evaluation form for survey questionnaire

1. Basic information:

Name:	Gender:
Professional Title:	Position:
Field of Study:	Affiliation:

2. Validity test (Please mark “ √ ” in the appropriate box corresponding to your assessment level)

2.1 How do you evaluate the overall design of the questionnaire?

Very Satisfied	Satisfied	Fairly Satisfied	Dissatisfied	Very Dissatisfied

2.2 How do you assess the structural design of the questionnaire?

Very Satisfied	Satisfied	Fairly Satisfied	Dissatisfied	Very Dissatisfied

2.3 How do you rate the content design of the questionnaire?

Very Satisfied	Satisfied	Fairly Satisfied	Dissatisfied	Very Dissatisfied

Results of validity test for physical fitness evaluation index selection questionnaire (n=12)

1. Overall questionnaire design test results

	Very Satisfied	Satisfied	Fairly Satisfied	Dissatisfied	Very Dissatisfied
Number	6	5	1		
Percentage	50.00%	41.67%	8.33%		

2. Questionnaire structure design test results

	Very Satisfied	Satisfied	Fairly Satisfied	Dissatisfied	Very Dissatisfied
Number	5	7			
Percentage	41.67%	58.33%			

3. Questionnaire content design test results

	Very Satisfied	Satisfied	Fairly Satisfied	Dissatisfied	Very Dissatisfied
Number	6	5	1		
Percentage	50.00%	41.67%	8.33%		
