# Supplementary material for: Physical fitness characteristics of elite freestyle skiing aerials athletes
Source: PLoS One. 2024 Jun 6;19(6):e0304912. doi: 10.1371/journal.pone.0304912 (PMC11156283; doi:10.1371/journal.pone.0304912)
Supplement: S5 Appendix — (PDF) [file pone.0304912.s005.pdf]

Appendix 5-1

Table 1 Results of Shapiro-Wilk Normality Test for Body Morphology Indexes

Index	Male				Female			
	**Group**	**Statistic**	**Degrees of Freedom**	**Significance**	**Group**	**Statistic**	**Degrees of Freedom**	**Significance**
Height (cm)	1	0.759	6	**0.024***	1	0.909	5	0.464
	2	0.951	9	0.704	2	0.892	9	0.211
Body weight (kg)	1	0.879	6	0.264	1	0.935	5	0.628
	2	0.980	9	0.964	2	0.772	9	**0.010***
Upper limb length(cm)	1	0.951	6	0.747	1	0.729	5	**0.019***
	2	0.839	9	0.056	2	0.881	9	0.159
Lower limb length(cm)	1	0.817	6	0.084	1	0.874	5	0.281
	2	0.831	9	0.096	2	0.956	9	0.750
Achilles tendon length(cm)	1	0.958	6	0.801	1	0.878	5	0.302
	2	0.873	9	0.134	2	0.947	9	0.658
Shoulder width(cm)	1	0.727	6	**0.012***	1	0.920	5	0.527
	2	0.939	9	0.569	2	0.931	9	0.493
Pelvis width(cm)	1	0.812	6	0.075	1	0.923	5	0.546
	2	0.955	9	0.742	2	0.822	9	**0.036***
Thigh circumference (cm)	1	0.899	6	0.367	1	0.900	5	0.411
	2	0.921	9	0.401	2	0.884	9	0.174
Calf circumference (cm)	1	0.863	6	0.198	1	0.944	5	0.696
	2	0.916	9	0.356	2	0.883	9	0.113
Waist circumference (cm)	1	0.964	6	0.850	1	0.889	5	0.350
	2	0.897	9	0.234	2	0.825	9	0.039
Fat-free body weight (kg)	1	0.866	6	0.209	1	0.827	5	0.131
	2	0.959	9	0.783	2	0.940	9	0.583
Body fat percentage (%)	1	0.929	6	0.569	1	0.930	5	0.596
	2	0.799	9	**0.029***	2	0.839	9	0.056
Body mass index	1	0.938	6	0.642	1	0.914	5	0.494
	2	0.849	9	0.072	2	0.924	9	0.427
Waist-hip ratio	1	0.858	6	0.181	1	0.828	5	0.135
	2	0.818	9	**0.033***	2	0.927	9	0.456
Quetelet index	1	0.917	6	0.482	1	0.717	5	**0.014***
	2	0.949	9	0.674	2	0.832	9	**0.047***

Note: The elite group is denoted as 1, while the general group is denoted as 2. Significance level markers: *indicates P < 0.05, ** indicates P < 0.01. The same notation applies to subsequent charts.

Table 2 Results of Shapiro-Wilk Normality Test for Physiological Function Indexes

Index	Male				Female			
	**Group**	**Statistic**	**Degrees of Freedom**	**Significance**	**Group**	**Statistic**	**Degrees of Freedom**	**Significance**
Maximum anaerobic power(w)	1	0.843	6	0.137	1	0.949	5	0.728
	2	0.903	9	0.271	2	0.863	9	0.104
Relative maximum anaerobic power(w/kg)	1	0.919	6	0.498	1	0.878	5	0.300
	2	0.959	9	0.784	2	0.847	9	0.069
Maximal oxygen intake(L/min)	1	0.857	6	0.180	1	0.997	5	0.997
	2	0.778	9	**0.011***	2	0.934	9	0.517
Relative maximum oxygen uptake(L/kg/min)	1	0.840	6	0.130	1	0.909	5	0.463
	2	0.958	9	0.777	2	0.967	9	0.864
Hemoglobin(g/L)	1	0.949	6	0.734	1	0.825	5	0.128
	2	0.923	9	0.420	2	0.990	9	0.996
Red-cell count(10^{12}/L)	1	0.945	6	0.699	1	0.861	5	0.232
	2	0.972	9	0.908	2	0.972	9	0.910
Bloodurea (mmol/L)	1	0.976	6	0.929	1	0.961	5	0.816
	2	0.946	9	0.650	2	0.877	9	0.146
Serum testosterone (ug/dl)	1	0.833	6	0.115	1	0.984	5	0.955
	2	0.948	9	0.668	2	0.917	9	0.371
Serum cortisol(ug/dl)	1	0.953	6	0.767	1	0.970	5	0.873
	2	0.947	9	0.654	2	0.905	9	0.280

Table 3 Results of Shapiro-Wilk Normality Test for Physical Quality Indexes

Index	Male				Female			
	Group	Statistic	Degrees of Freedom	Significance	Group	Statistic	Degrees of Freedom	Significance
Barbell squat（kg）	1	0.907	6	0.415	1	0.828	5	0.133
	2	0.798	9	**0.020***	2	0.890	9	0.201
Pull up（repetitions）	1	0.837	6	0.122	1	0.894	5	0.377
	2	0.929	9	0.467	2	0.875	9	0.137
Backward ball toss (m)	1	0.848	6	0.152	1	0.878	5	0.299
	2	0.923	9	0.418	2	0.921	9	0.397
Leftward ball toss (m)	1	0.907	6	0.414	1	0.788	5	0.065
	2	0.931	9	0.493	2	0.940	9	0.587
Rightward ball toss (m)	1	0.976	6	0.930	1	0.945	5	0.703
	2	0.966	9	0.861	2	0.976	9	0.943
Overhead barbell squat on balance pads (s)	1	0.894	6	0.339	1	0.872	5	0.277
	2	0.883	9	0.167	2	0.923	9	0.420
Quick v-up (s)	1	0.973	6	0.909	1	0.964	5	0.833
	2	0.950	9	0.686	2	0.675	9	**0.001****
Three-step stable jump with left leg (m)	1	0.945	6	0.699	1	0.930	5	0.599
	2	0.931	9	0.492	2	0.877	9	0.147
Three-step stable jump of right leg (m)	1	0.976	6	0.930	1	0.988	5	0.974
	2	0.966	9	0.861	2	0.827	9	**0.041***
Standing long jump (m)	1	0.899	6	0.368	1	0.889	5	0.352
	2	0.846	9	0.067	2	0.967	9	0.868
Power clean (kg)	1	0.983	6	0.966	1	0.735	5	**0.021***
	2	0.912	9	0.327	2	0.886	9	0.180
30-meter sprint (s)	1	0.912	6	0.453	1	0.903	5	0.426
	2	0.868	9	0.117	2	0.854	9	0.083
12-minute run (m)	1	0.882	6	0.280	1	0.865	5	0.249
	2	0.925	9	0.432	2	0.917	9	0.371
Agile running (s)	1	0.970	6	0.893	1	0.930	5	0.598
	2	0.909	9	0.306	2	0.945	9	0.631
Barbell squat/Weight	1	0.901	6	0.381	1	0.876	5	0.291
	2	0.860	9	0.097	2	0.975	9	0.930
Power clean/Weight	1	0.926	6	0.551	1	0.992	5	0.987
	2	0.911	9	0.321	2	0.965	9	0.847

Appendix 5-2

Table 1 Comparison of Male Athletes' Body Morphology Indexes between Elite and General Groups

	**Independent Samples T-Test (M±SD)**					**Non-parametric test : Median (Q1-Q3)**				
**Index**	**Elite group （n=6）**	**General group （n=9）**	**T**	**P**	**d**	**Elite group （n=6）**	**General group （n=9）**	**Z**	**P**	**r**
Height (cm)						175 (172.93~175.25)	175 (172.50~178.30)	-0.487	0.626	-0.12
Body weight (kg)	68.57±3.51	69.21±4.82	-0.291	0.776	-0.15					
Upper limb length (cm)	72.06±1.36	72.69±1.31	-0.897	0.386	-0.47					
Lower limb length (cm)	101.73±2.03	102.42±2.53	-0.562	0.584	-0.30					
Achilles tendon length(cm)	22.17±1.04	21.79±0.73	0.837	0.481	0.42					
Shoulder width(cm)						45.42 （43.77~46.16）	42.43 （40.64~44.55）	-1.650	0.990	-0.43
Pelvis width (cm)	29.70±2.17	29.84±2.01	-0.126	0.902	-0.67					
Thigh circumference (cm)	54.23±1.61	51.73±2.14	2.430	0.030*	1.32					
Calf circumference (cm)	37.51±1.63	35.19±1.94	2.391	0.032*	1.29					
Waist circumference (cm)	73.58±2.59	70.29±2.82	2.291	0.039*	1.22					
Fat-free body weight(kg)	60.34±1.70	57.79±2.67	2.304	0.038*	1.14					
Body fat percentage (%)						11.45 （9.43~13.08）	16.40 (13.35~17.75)	-2.593	0.010*	-0.67
Body mass index	22.62±1.11	22.46±1.28	0.252	0.805	0.13					
Waist-hip ratio						0.80 （0.79~0.83）	0.82 （0.80~0.83）	-0.773	0.440	-0.20
Quetelet index	393.70±19.05	394.19±22.65	-0.043	0.966	-0.02					

Table 2 Comparison of Female Athletes' Body Morphology Indexes between Elite and General Groups

	Independent Samples T-Test (M±SD)					Non-parametric test : Median (Q1-Q3)				
Index	Elite group (n=5)	General group (n=9)	T	P	d	Elite group (n=5)	General group (n=9)	Z	P	r
Height (cm)	161.90±3.94	158.78±3.46	1.54	0.149	0.84					
Body weight (kg)						55.60 (53.50~59.25)	54.20 (51.55~56.95)	-0.934	0.350	-0.25
Upper limb length (cm)						68.10 (64.52~68.83)	66.55 (65.07~68.21)	-1.000	0.317	-0.27
Lower limb length(cm)	93.65±3.12	93.47±2.34	0.123	0.904	0.07					
Achilles tendon length(cm)	20.64±0.68	20.41±0.75	0.567	0.851	0.32					
Shoulder width(cm)	40.16±2.21	41.02±1.79	-0.795	0.471	-0.43					
Pelvis width (cm)						25.98 (25.72~26.98)	25.72 (25.44~26.75)	-1.133	0.257	-0.30
Thigh circumference (cm)	54.54±1.93	50.25±3.71	2.382	0.035 *	1.45					
Calf circumference (cm)	35.06±0.68	34.18±1.61	1.150	0.273	0.71					
Waist circumference (cm)	71.32±2.55	67.35±1.32	3.243	0.021 *	1.96					
Fat-free body weight(kg)	45.48±2.71	42.33±1.98	2.509	0.027*	1.33					
Body fat percentage (%)	19.12±2.79	23.54±6.68	-1.395	0.188	-0.86					
Body mass index	21.60±0.87	21.94±1.90	-0.377	0.713	-0.23					
Waist-hip ratio	0.82±0.01	0.84±0.05	-0.666	0.518	-0.55					
Quetelet index						341.88 (338.18~358.56)	345.22 (322.29~361.75)	-0.200	0.841	-0.05

Table 3 Comparison of Physiological Function Indexes of Male Athletes in Elite and General Groups

Index	Independent Samples T-Test (M±SD)					Non-parametric test : Median (Q1-Q3)				
	Elite group (n＝6)	General group (n＝9)	T	P	d	Elite group (n＝6)	General group (n＝9)	Z	P	r
Maximum anaerobic power(w)	1094.25±54.58	981.86±31.84	5.068	0.001* *	2.52					
Relative maximum anaerobic power(w/kg)	16.00±1.31	14.24±1.05	2.882	0.013*	1.48					
Maximal oxygen intake(L/min)						3.66 (3.51~3.76)	3.60 (3.53~3.67)	-1.122	0.262	0.29
Relative maximum oxygen uptake(ml/kg/min)	53.00±5.32	51.92±5.11	0.397	0.698	0.21					
Hemoglobin(g/L)	149.67±8.12	152.89±7.56	-0.786	0.446	-0.41					
Red-cell count(10^{12}/L)	5.11±0.25	5.36±0.27	-1.750	0.104	-0.96					
Bloodurea (mmol/L)	5.48±1.03	5.04±0.99	0.831	0.421	0.44					
Serum testosterone (ug/dl)	722.28±89.40	668.54±150.20	0.783	0.448	0.44					
Serum cortisol(ug/dl)	13.21±0.68	13.54±1.58	-0.471	0.645	-0.27					

Table 4 Comparison of Physiological Function Indexes of Female Athletes in Elite and General Groups

Index	Independent Samples T-Test (M±SD)				
	Elite group （n＝5）	General Group （n＝9）	T	P	d
Maximum anaerobic power(w)	822.61±64.27	742.12±48.43	2.662	0.031*	1.41
Relative maximum anaerobic power(w/kg)	14.62±0.35	13.45±0.69	3.48	0.005* *	2.14
Maximal oxygen intake(L/min)	2.64±0.21	2.56±0.22	0.665	0.518	0.37
Relative maximum oxygen uptake(ml/kg/min)	47.06±3.70	46.54±4.48	0.223	0.819	0.13
Hemoglobin(g/L)	129.20±8.84	126.00±6.96	0.75	0.467	0.40
Red-cell count(10^{12}/L)	4.33±0.24	4.53±0.25	-1.43	0.177	-0.82
Blood urea(mmol/L)	4.62±0.80	4.31±0.87	0.65	0.133	0.37
Serum testosterone(ug/dl)	61.35±19.29	45.90±16.04	1.61	0.527	0.87
Serum cortisol(ug/dl)	14.36±2.10	14.93±2.06	-0.49	0.632	-0.27

Table 5 Comparison of Physical Quality Indexes of Male Athletes in Elite and General Groups

	Independent Samples T-Test (M±SD)					Non-parametric test : Median (Q1-Q3)				
**Index**	**Elite group （n＝6）**	**General group （n＝9）**	**T**	**P**	**d**	**Elite group （n＝6）**	**General group （n＝9）**	**Z**	**P**	**r**
Barbell squat（kg）						160 （147.50~170）	140 （130~145）	-2.280	0.023 *	-0.59
Pull up （repetitions）	19.50±5.28	14.00±3.39	2.473	0.028*	1.24					
Backward ball toss (m)	6.19±0.71	5.41±0.59	2.303	0.038*	1.19					
Leftward ball toss (m)	6.87±0.67	6.03±0.70	2.310	0.038*	1.23					
Rightward ball toss (m)	7.36±0.44	6.80±0.35	2.733	0.017*	1.41					
Overhead barbell squat on balance pads (s)	9.7±1.00	11.71±1.75	-2.532	0.025*	-1.41					
Quick v-up (s)	9.73±0.54	9.80±0.57	-0.210	0.837	-0.13					
Power clean (kg)	100.08±7.00	79.78±21.75	2.188	0.048*	1.26					
Standing long jump (m)	275.00±9.48	260.67±13.50	2.244	0.043*	1.23					
Three-step stable jump with left leg (m)	7.31±0.41	6.75±0.44	2.482	0.028*	1.32					
Three-step stable jump of right leg (m)	7.36±0.44	6.80±0.35	2.733	0.017*	1.41					
30-meter sprint (s)	3.98±0.16	4.72±0.79	-2.202	0.046*	-1.30					
12-minute run (m)	2728.33 ±248.61	2587.78 ±213.17	1.172	0.262	0.61					
Agile running (s)	6.40±0.34	6.80±0.12	-3.332	0.005**	-1.57					
Barbell squat/Weight	2.29±0.25	2.03±0.20	2.191	0.047*	1.15					
Power clean/Weight	1.46±0.13	1.14±0.29	2.482	0.027*	1.42					

Table 6 Comparison of Physical Quality Indexes of Female Athletes in Elite Group and General Groups

	Independent Samples T-Test (M±SD)					Non-parametric test : Median (Q1-Q3)				
**Index**	**Elite group (n＝5)**	**General group (n＝9)**	**T**	**P**	**d**	**Elite group (n＝5)**	**General group (n＝9)**	**Z**	**P**	**r**
Barbell squat （kg）	101.00±12.44	69.77±9.23	5.375	0.001* *	2.85					
Pull up （repetitions）	10.20±1.79	6.33±4.64	2.222	0.048*	1.10					
Backward ball toss (m)	4.69±0.36	4.04±0.54	2.374	0.035*	1.42					
Leftward ball toss (m)	5.23±0.92	4.32±0.60	2.249	0.044*	1.17					
Rightward ball toss (m)	5.22±0.90	4.22±0.57	2.565	0.025*	1.33					
Overhead barbell squat on balance pads (s)	10.78±1.35	16.93±4.39	-3.002	0.011*	-1.89					
Quick v-up (s)						10.29 （9.91~10.77）	10.11 （9.78~11.11）	-0.134	0.894	-0.04
Power clean (kg)						60 （55~65）	40 （30~42.5）	-3.004	0.002 * *	-0.80
Standing long jump (m)	226.20±11.69	201.56±12.20	3.672	0.003* *	2.06					
Three-step stable jump with left leg(m)	5.91±0.33	5.05±0.80	2.261	0.043*	1.41					
Three-step stable jump of right leg (m)						6.00 （5.75~6.32）	5.50 （3.57~5.76）	-2.403	0.016*	-0.64
30-meter sprint (s)	4.69±0.13	5.13±0.41	-2.962	0.014*	-1.45					
12-minute run(m)	2304±269.19	2507.78 ±260.37	-1.387	0.191	-0.77					
Agile running(s)	6.96±0.51	7.74±0.46	-2.917	0.013*	-1.61					
Barbell squat/Weight	1.80±0.19	1.28±0.24	4.143	0.001* *	2.40					
Power clean/Weight	1.05±0.13	0.64±0.16	4.952	0.001* *	2.81					
